# A Meta-Learner Framework to Estimate Individualized Treatment Effects for Survival Outcomes

**DOI:** 10.6339/24-jds1119

**Published:** 2024-02-05

**Authors:** Na Bo, Yue Wei, Lang Zeng, Chaeryon Kang, Ying Ding

**Affiliations:** 1 Department of Biostatistics, University of Pittsburgh, U.S.A; 2 Novartis, China

**Keywords:** age-related macular degeneration, individualized treatment effect, precision medicine, randomized clinical trials, survival outcomes

## Abstract

One crucial aspect of precision medicine is to allow physicians to recommend the most suitable treatment for their patients. This requires understanding the treatment heterogeneity from a patient-centric view, quantified by estimating the individualized treatment effect (ITE). With a large amount of genetics data and medical factors being collected, a complete picture of individuals’ characteristics is forming, which provides more opportunities to accurately estimate ITE. Recent development using machine learning methods within the counterfactual outcome framework shows excellent potential in analyzing such data. In this research, we propose to extend meta-learning approaches to estimate individualized treatment effects with survival outcomes. Two meta-learning algorithms are considered, T-learner and X-learner, each combined with three types of machine learning methods: random survival forest, Bayesian accelerated failure time model and survival neural network. We examine the performance of the proposed methods and provide practical guidelines for their application in randomized clinical trials (RCTs). Moreover, we propose to use the Boruta algorithm to identify risk factors that contribute to treatment heterogeneity based on ITE estimates. The finite sample performances of these methods are compared through extensive simulations under different randomization designs. The proposed approach is applied to a large RCT of eye disease, namely, age-related macular degeneration (AMD), to estimate the ITE on delaying time-to-AMD progression and to make individualized treatment recommendations.

## 1 Introduction

Traditional clinical studies often prioritize estimating the average treatment effect across the study population. While this provides valuable insights, it can overlook a crucial aspect of patient care: treatment heterogeneity based on individual patient’s characteristics. Treatment heterogeneity refers to the variations in treatment effects among patients due to their unique genetic makeup, medical history, and other individual factors. Failing to consider this can have significant implications for patient care. Precision medicine aims to provide patients with the most suitable treatment option, considering their unique genetic and clinical characteristics. This requires a deep understanding of estimating the treatment effect given individual patient’s covariates, referred to as estimating the individualized treatment effect (ITE).

Recent advancements in statistical methodologies for estimating ITE have primarily focused on continuous or binary outcomes. For example, leveraging the benefits of tree structures to interpret outcome estimations based on individual characteristics, Hill (2011) proposed a Bayesian Additive Regression Trees (BART) approach to estimate ITE. Wager and Athey (2018) introduced a causal forest (CF) method by modifying the splitting rule in standard random forest algorithms to directly maximize the treatment effect within each node. More recently, Koch et al. (2023) reviewed four approaches that estimate ITE through neural network architectures: TARNet learns ITE by balancing covariates between treatment and control groups through representation learning (Shalit et al., 2017); CFRNet, an extension of TARNet that incorporates an integral probability metric (Johansson et al., 2022); Dragonnet, another extension of TARNet with the inclusion of inverse probability weighting (Shi et al., 2019); and GANITE, which learns ITE through adversarial training (Yoon et al., 2018). Nevertheless, these approaches are formulated through a specific algorithm (e.g., trees or neural networks), lacking the flexibility to choose any desired parametric or nonparametric models for estimating ITE. On the other hand, meta-learning algorithms, another popular framework, provide flexible choices of parametric or nonparametric methods (e.g., linear regression, Lasso, random forests, neural networks, etc.) to estimate ITE. For example, S-learner and T-learner were developed from the “virtual twins” (VT) approach (Foster et al., 2011) to model the ITE by predicting response probabilities for treatment and control “twins” for each subject and identify individuals who can benefit from the treatment over a control therapy. Kunzel et al. (2018) extended it to X-learner which provides a more accurate ITE estimation when sample sizes are (very) different in two treatment arms. Theoretical properties of meta-learner methods have also been studied (Kunzel et al., 2018; Curth and van der Schaar, 2021; Kennedy, 2023).

While methods and their properties for estimating ITEs with continuous or binary outcomes have been extensively examined, fewer studies exist for survival outcomes. Survival outcomes are of primary interest in clinical trials for cancer and progressive diseases. One challenge specific to analyzing survival outcomes is the presence of censoring. Several recent publications have introduced novel methods of estimating ITE with survival outcomes. For example, Zhu and Gallego (2020) introduced a new framework to estimate individualized hazard function through targeted maximum likelihood estimation (TMLE) and then used a scoring system to identify features that contribute to heterogeneous ITEs. However, their approach requires converting the original survival data into discretized times. In a different approach, Cui et al. (2023) leveraged the interpretation of ITEs from tree structures to develop a causal survival forest (CSF) method, extending the original causal forests to handle right-censored data. On the other hand, Tabib and Larocque (2020) proposed a special splitting rule in the traditional random forests to maximize the distance between the ITE of the left and right node populations. In their method, the treatment effect is measured as the difference between the mean survival time of a treated subject and that of a control subject. While these methods can accommodate right-censored data, they either estimate ITE using discretized times or rely on a specific algorithm such as the tree-based methods. Such limitations restrict the flexibility in applying any desired model to estimate ITE, especially when dealing with large-scale data such as genetics data.

In this study, we present a novel extension of two existing meta-learning ideas, namely T-learner and X-learner, to estimate ITE in randomized clinical trials (RCTs) with right-censored survival outcomes. The motivation behind this project stems from investigating the treatment effects on delaying the progression of a serious eye disease called age-related macular degeneration (AMD). AMD is a polygenic and progressive neurodegenerative disorder that can result in blindness among the elderly population. Advancements in genotyping technologies have facilitated the collection and analysis of single nucleotide polymorphisms (SNP) data to identify potential genetic risk factors associated with time-to-AMD progression (Yan et al., 2018; Sun and Ding, 2019). The antioxidants plus zinc treatment (AREDS formula) is considered a potential treatment in delaying AMD progression and its average treatment effect on time-to-AMD-progression has been studied through the Age-Related Eye Disease Study (AREDS) (, 1999), a large multi-center RCT. Although some studies have attempted to examine genetic risk factors associated with treatment heterogeneity, the findings are controversial (Cascella et al., 2018). For instance, while Klein et al. (2008) and Seddon et al. (2016) reported differential responses in the *CFH* and *ARMS2* gene regions to AREDS formula, Chew et al. (2015) found no such differential responses after controlling for multiple testing. To our best knowledge, there is no existing work that quantified the ITE of AREDS formula on time-to-AMD progression. In this paper, we define the ITE to be the difference in progression-free probabilities if receiving the AREDS formula vs if receiving a placebo, given individual patient’s characteristics. We implement and evaluate the performance of three different types of machine learning methods accompanied with two meta-learning algorithms in estimating ITEs under various randomization designs and compare them with an existing method, causal survival forests. Based on the ITE estimates, we provide treatment recommendations for each individual and identify genetic risk factors contributing to treatment heterogeneity.

Our contributions are in three folds. First, we extend the meta-learning algorithms to right-censored data by appropriately addressing the challenges unique to survival outcomes. Secondly, we extensively study the performance of meta-learning algorithms when implemented with different types of machine-learning methods and provide practical guidelines for their applications in RCTs. Finally, we apply the top performed methods on the motivating study, and successfully identify and validate the genetic risk factors that are predictive of ITEs in time-to-AMD progression, which is the first analysis to quantify ITEs of the AREDS formula.

The paper is organized as follows. The settings, assumptions, and proposed methods are described in Section 2. In Section 3, we present comprehensive simulations to compare the performance of different methods in estimating ITEs and providing treatment recommendations under various scenarios. The results of the application of the proposed methods in analyzing the AREDS study are reported in Section 4. Finally, we discuss the potential limitations of the proposed methods and future research directions in Section 5.

## 2 Method

### 2.1 Notation

The methods we consider are applicable to either a simple RCT or a stratified RCT in which randomization depends on baseline covariates. We consider a two-treatment arm comparison, denoted by Z=1 for treatment and Z=0 for control. X is a d-dimensional vector of covariates. T and C denote the survival time and censoring time, respectively. Then the observed time is Y=min(T,C), with the event indicator δ=I(T⩽C). Let (T(0),T(1)) denote the potential survival times under Neyman-Rubin’s potential outcome framework (Rubin, 1974; Splawa-Neyman et al., 1990), indicating the survival times if the patient receives control and treatment, respectively. Similarly, (δ(0),δ(1)) indicates the event indicator, and (C(0),C(1)) indicates the censoring time under control and treatment. We assume the non-informative censoring assumption under which the censoring time is independent of the survival time given covariates and treatment assignment: C(z)⫫T(z)∣(X,Z). Before defining ITE, we first define the conditional average treatment effect (CATE) function given patient characteristics following Neyman-Rubin’s potential outcome framework:

(1) $$\theta (t,x)=E\{I(T(1)>t|X=x)\}-E\{I(T(0)>t|X=x)\}\;=E\{I(T>t|Z=1,X=x)\}-E\{I(T>t|Z=0,X=x)\}\;=S_{1}(t|x)-S_{0}(t|x),$$

where $S_{z}(t|x)=S(t|Z=z,X=x)=P(T>t|Z=z,X=x)$ with z=1 or 0 denotes the conditional survival function given covariates X=x for treatment arm z. The time t in the CATE function is pre-chosen. For example, one can use the median or mean survival time as this t. For a specific patient i with characteristics $x_{i}$, we plug $x_{i}$ into the CATE function and define their ITE on the survival probability scale as:

(2) $$\theta_{i}\left(t\right)=S_{1}\left(t|x_{i}\right)-S_{0}\left(t|x_{i}\right).$$

Note that the second line of definition (1) is guaranteed by the commonly assumed three assumptions in causal inference: consistency, unconfoundedness, and positivity. Consistency assumption guarantees the counterfactual model applied to the observed outcomes as Y=ZY(1)+(1−Z)Y(0) and δ=Zδ(1)+(1−Z)δ(0). The unconfoundedness assumption rules out the existence of unobserved factors that affect treatment choice and correlate with the outcome, which is Z⫫(T(0),T(1))∣X. The positivity assumption requires that for each value of covariates, there is a positive probability of being assigned to both treatments: P(Z=1∣X=x)∈(0,1). Equivalently, this assumes that there is sufficient overlap in the characteristics of treated and control patients.

### 2.2 Estimating ITE with Meta-Learning Algorithms

In this study, inspired by Kunzel et al. (2018), we propose to extend two meta-algorithms to estimate ITEs with survival outcomes by estimating the CATE function in (1).

#### T-learner (plug-in estimator (Curth and van der Schaar, 2021))

T-learner fits two separate prediction models of conditional survival function using patients under each treatment arm, denoted by ${\overset{ˆ}{S}}_{1}(t|X)$ and ${\overset{ˆ}{S}}_{0}(t|X)$. They are also called “base learners”. Besides fitting parametric models for the survival functions, machine learning methods can be used to build the base learners, which are typically preferred especially with large-scale X. In the T-learner, the treatment indicator is no longer a covariate in the prediction model. The ITE estimate for a specific patient i with characteristics $X=x_{i}$ is given by

(3) $${\overset{ˆ}{\theta }}_{i}(t)=\overset{ˆ}{\theta }\left(t|X=x_{i}\right)={\overset{ˆ}{S}}_{1}\left(t|x_{i}\right)-{\overset{ˆ}{S}}_{0}\left(t|x_{i}\right).$$

#### X-learner

X-learner is a two-step approach. The first step is to perform T-learner, that is, to construct a T-learner type of estimated ITE for each patient. In the second step, we further regress these T-learner estimated ITEs on the covariates using patients in the treatment arm or control arm separately. The final ITE is a weighted average of the two ITE estimates from these separate regressions in step 2. The detailed implementation steps are provided below.

*Step 1*. Compute the T-learner type of ITE estimates ${\tilde{D}}_{i}(t)$ for each patient by taking the difference of the predicted conditional survival probabilities under treatment and under control:
$${\tilde{D}}_{i}(t)={\overset{ˆ}{S}}_{1}\left(t|x_{i}\right)-{\overset{ˆ}{S}}_{0}\left(t|x_{i}\right),$$
  where “base learners” ${\overset{ˆ}{S}}_{1}(t|x)$ and ${\overset{ˆ}{S}}_{0}(t|x)$ are built using patients in the treatment arm and control arm respectively. Note that ${\tilde{D}}_{i}(t)$ is essentially the same as ${\overset{ˆ}{\theta }}_{i}(t)$ from equation (3).
*Step 2*. Regress ${\tilde{D}}_{i}(t)$ on covariates $x_{i}$ using patients in treatment and control arm separately, to estimate two CATE functions ${\overset{ˆ}{\theta }}_{z}(t,x)$:
$${\overset{ˆ}{\theta }}_{z=1}\left(t,x\right):ℳ\left(\tilde{D}\left(t\right)\sim x;z=1\right),\;{\overset{ˆ}{\theta }}_{z=0}\left(t,x\right):ℳ\left(\tilde{D}\left(t\right)\sim x;z=0\right),$$
  where ℳ denotes a fitted model using data $\left({\tilde{D}}_{i}(t),x_{i}\right)$.

For each patient, we estimate their ITE twice, one based on the estimated CATE function using treated patients (i.e., ${\overset{ˆ}{\theta }}_{z=1}\left(t,x_{i}\right)$), and the other based on the estimated CATE function using control patients (i.e., ${\overset{ˆ}{\theta }}_{z=0}\left(t,x_{i}\right)$). The final ITE estimate is given by a weighted average of these two estimates:

(4) $${\overset{ˆ}{\theta }}_{i}(t)=g\left(x_{i}\right){\overset{ˆ}{\theta }}_{z=0}\left(t,x_{i}\right)+\left\{1-g\left(x_{i}\right)\right\}{\overset{ˆ}{\theta }}_{z=1}\left(t,x_{i}\right),$$

where g(x)∈(0,1) is a weight function. Following the recommendation of Kunzel et al. (2018), one may use the known randomization probability p as the weight (g(x)=p) if it is a RCT with p probability to be randomized to the treatment arm or the estimated propensity score as the weight $g(x)=\overset{ˆ}{P}(Z=1|X=x)$ if the randomization scheme is more complex (depends on covariates) with propensity score function unknown.

Here, we explain the difference between the original X-learner for continuous or binary data and the proposed extension to survival data. In the first step of the original X-learner, it constructs the ITE by the difference between the observed outcome (factual outcome) the predicted survival probability (counterfactual outcome) for each patient, denoted by ${\tilde{D}}_{i}(t)=I\left(T_{i}>t\right)-{\overset{ˆ}{S}}_{0}\left(t|x_{i}\right)$ if patient i is in the treatment arm and ${\tilde{D}}_{i}(t)={\overset{ˆ}{S}}_{1}\left(t|x_{i}\right)-I\left(T_{i}>t\right)$ if patient i is in the control arm. However, this original construction cannot be directly applied to censored data as $I\left(T_{i}>t\right)$ is unknown for those patients who are censored before the pre-specified time t. Thus, we propose to use both predicted conditional survival probabilities to calculate this quantity ${\tilde{D}}_{i}(t)$ for each subject, which means that both factual and counterfactual outcomes are estimated. As demonstrated in the original paper (Kunzel et al., 2018), X-learner has advantages when sample sizes are (very) different between two arms and/or when randomization depends on baseline covariates. This is achieved through the weight function g(x). The performance of both T- and X-learners on survival data will be examined through simulations in Section 3.

Note that the estimation of ITE heavily relies on the performance of the base learners. In this paper, we investigate three types of machine learning methods to build base learners for survival data: random survival forest (RSF) (Ishwaran and Kogalur, 2007), Bayesian accelerated failure time model (BAFT) (Henderson et al., 2020), and deep survival neural network (DNNSurv) (Sun et al., 2020).

### 2.3 Identification of Important Variables

As ITE estimates characterize the treatment heterogeneity, they can be used to guide treatment recommendations for each individual. From equation (2), a positive value of $\theta_{i}(t)$ indicates that the survival probability under Z=1 is larger than that under Z=0 for a patient i with $X=x_{i}$. Therefore, a possible approach is to use 0 as the threshold value to recommend treatment choices based on the estimated ${\text{ITE}:\;\text{RTC}}_{i}=1\left\{{\overset{ˆ}{\theta }}_{i}(t)>0\right\}$ (‘RTC’ stands for recommended treatment choice). That is, the treatment (Z=1) is recommended for individuals whose estimated ITE is positive; and on the contrary, the control (Z=0) is recommended for individuals whose estimated ITE is non-positive. In this situation with only two treatments to consider, the treatment recommendation becomes a binary classification problem. The important features are defined as those that contribute most to reducing the classification error. We will use the Boruta algorithm (Kursa and Rudnicki, 2010) to identify these important features.

The Boruta algorithm first generates “shadow variables” by shuffling the values of each original feature to remove any potential correlation with the target binary outcome. These “shadow variables” are then added to the original dataset so that the total number of features is doubled. A random forest classifier is used on the new dataset, and z-scores are computed for all features, including real and shadow ones. The maximum score for all “shadow variables” serves as a threshold to assign a “Hit” to the original features (i.e., if the z-score of original features is greater than the threshold, a “Hit” is marked). For variables with undetermined importance, a two-sided test of equality with the max z-score in the shadow features is conducted. Those with significantly lower z-score are permanently removed from the dataset. After removing unimportant features and all shadow variables, repeat the procedure until the importance is assigned for all features or the pre-specified number of iterations is reached. This algorithm identifies variables that significantly contribute to the treatment recommendation rule. We can better understand the heterogeneity in the treatment efficacy from these important features, which can guide future trial designs.

## 3 Simulations

We conducted comprehensive simulations to evaluate the finite sample performance of the proposed methods for estimating ITE under various RCT settings.

### 3.1 Simulation Design

We simulated true survival times from parametric regression models with two types of error distributions: Weibull distribution and Log-logistic distribution with nonzero treatment effect (i.e., the CATE function θ(t,x)≠0). We simulated data from Weibull regression because Weibull satisfies both proportional hazards (PH) and accelerated failure model (AFT) assumptions, which allows us to have more flexibility in choosing machine learning methods for base learners; while Log-logistic distribution only belongs to the AFT model family, which will be used to examine the robustness of the proposed methods when some modeling assumptions of base learners are not met. Among the base learners we evaluated, RSF and DNNSurv assume the PH property in their models, while BAFT assumes the AFT property.

Ten covariates $X_{1},\ldots ,X_{10}$ were simulated independently from $N\left(0,0.35^{2}\right)$ and then three binary covariates were generated by ${\tilde{X}}_{j}=1\left\{X_{j}>0\right\}$ for j=8,9,and10. The treatment indicator Z was generated from Bernoulli(p). We considered three randomization designs: (1) a balanced design where p=0.5; (2) an extremely unbalanced design where p=0.05; and (3) a dependent design with $\text{logit}(p|X)=\beta_{0}+1.3X_{1}-0.8X_{5}.\;\beta_{0}$ was selected to have a 1:1 treatment-control ratio.

For the Weibull case, the true survival model is: $S_{z}(t|X)=S(t|X=x,Z=z)=\text{exp}\left\{-e^{h_{z}(x)}{\left(\frac{t}{\lambda_{z}}\right)}^{\eta }\right\}$. Then by inverse-transforming the survival function, the survival time T was simulated from: $T=\lambda_{z}{\left\{-\text{log}(U)/e^{h_{z}(x)}\right\}}^{\frac{1}{n}}$, where U~Unif[0,1]. We set the common shape parameter η=2 and two separate scale parameters $\lambda_{0}=18$ and $\lambda_{1}=20$ for control and treatment, respectively. The censoring time was simulated from an exponential distribution to have an overall censoring rate of 30%. Three types of heterogeneous treatment effects were generated through the following functions for $h_{z}(x)$:

$$\text{S1:}h_{0}(x)=0.2X_{1}+0.7X_{2}+0.4{\tilde{X}}_{9},\;h_{1}(x)=-0.5X_{1}-2X_{2}-0.25{\tilde{X}}_{9}.$$

$$\text{S2:}h_{0}(x)=-0.5X_{1}+0.7X_{2}+0.2{\tilde{X}}_{9}+0.9X_{2}{\tilde{X}}_{9},\;h_{1}(x)=-0.05e^{X_{1}}-0.2X_{2}^{2}+0.35{\tilde{X}}_{9}.$$

$$\text{S3:}h_{0}(x)=-0.5X_{1}+0.7X_{2}+0.2{\tilde{X}}_{9}+0.9X_{2}{\tilde{X}}_{9}+0.6X_{3}-0.5X_{4}^{2}+0.6{\tilde{X}}_{8},\;h_{1}\left(x\right)=-0.05e^{X_{1}}-0.2X_{2}^{2}+0.2{\tilde{X}}_{9}-0.1e^{X_{5}}+0.7\;\text{sin}\;\left(X_{6}\right)+0.5{\tilde{X}}_{10}.$$

In Scenario 1, three common covariates are associated with the outcome through linear relationships in both treatment groups. In contrast, in Scenario 2, the same covariates are associated with the outcome through nonlinear relationships. In Scenario 3, different (with overlap) covariates are associated with the outcome through a complex nonlinear relationship in each treatment group.

For the Log-logistic case, the true survival model is: $S_{z}(t|X)=S(t|X=x,Z=z)=\frac{1}{1+\text{exp}\left\{\frac{\text{log}(t)-h_{z}(x)}{\sigma_{z}}\right\}}$. The scale parameter $\sigma_{z}$ was set to different values for different scenarios ($\sigma_{0}=2$ and $\sigma_{1}=1$ for Scenario 1 and $\sigma_{0}=1$ and $\sigma_{1}=0.5$ for Scenarios 2 and 3) to allow more than 50% individuals benefit from the treatment over control. The censoring time was also simulated from an exponential distribution to have an overall censoring rate of 30%. The covariates, treatment indicator, three randomization scenarios, and three types of heterogeneous treatment effects are the same as in the Weibull case.

We examined the following six combinations of two meta-algorithms and three base learners: RSF with T-learner (R-T), RSF with X-learner (R-X), BAFT with T-learner (B-T), BAFT with X-learner (B-X), DNNSurv with T-learner (D-T) and DNNSurv with X-learner (D-X). The true Weibull and Log-logistic regressions were also included to serve as the optimal case for the ITE estimate in two simulation designs. Under the scenarios when the true ITE was simulated from the Weibull regressions, we compared the proposed method with CSF (Cui et al., 2023), which is a “non-meta” type of method. CSF estimates CATE through orthogonal estimating equations. We trained each method on the training data of n=1,000 individuals and evaluated its performance on a large independent test data of $N=10^{5}$ individuals. In the second step of X-learner, any machine learning methods for continuous outcomes can be applied. We applied Gradient Boosting Machines (GBM) (Greenwell et al., 2020) in the second step to model $\tilde{D}(t)\sim x$ for each treatment arm separately. We built 1,000 boosting trees on each treatment arm using the following tuning parameters: the depth of each tree is 6, the minimum number of observations in the terminal nodes is 10, and the fraction of the training set observations randomly selected to propose the next tree in the expansion is 0.8. The weight function g(x) was estimated through the standard random forests (Liaw and Wiener, 2002). We chose the median survival time as our time of interest for constructing ITE. We also conducted a sensitivity analysis by choosing the 75% percentile of survival time as the time of interest for the Weibull case. We repeated the simulations for B=100 times for each setting and used the following metrics to evaluate the model performance.

### 3.2 Evaluation Metrics

To assess the ability of each method to uncover treatment heterogeneity, we evaluate their performance in estimating ITE within subgroups. We divided individuals into $G=\left\{G_{1},\ldots ,G_{Q}\right\}$ groups based on the Q quantiles of the true ITE values, and calculated bias and quantile-binned root mean squared error (RMSE), defined as:

$$\text{Bias}=\frac{1}{n}\sum_{i=1}^{n}\left({\overset{ˆ}{\theta }}_{i}(t)-\theta_{i}(t)\right),\;\text{RMSE}=\frac{1}{Q}\sum_{q=1}^{Q}\sqrt{\frac{1}{n_{q}}\sum_{i=1}^{n_{q}}{\left({\overset{ˆ}{\theta }}_{i}(t)-\theta_{i}(t)\right)}^{2}},$$

where $\theta_{i}(t)$ denotes the true ITE and ${\overset{ˆ}{\theta }}_{i}(t)$ denotes estimated ITE for individual i. We set Q=50 in all the simulations.

Since ITE estimates are used to make treatment recommendations, besides bias and RMSE, we further evaluate the ITE estimates from each method by overall accuracy (ACC), positive predictive value (PPV), negative predictive value (NPV), sensitivity, specificity, and F-score, as illustrated next. We assume that the treatment recommendation label based on the true ITE value is the gold standard: individuals with $\theta_{i}(t)>0$ should be recommended for treatment, whereas individuals with $\theta_{i}(t)⩽0$ should be recommended for control. Individuals with positive estimated ITE $\left({\overset{ˆ}{\theta }}_{i}(t)>0\right)$ were labeled as recommended for treatment (RT) and individuals with non-positive estimated ITE $\left({\overset{ˆ}{\theta }}_{i}(t)⩽0\right)$ were labeled as recommended for control (RC). Thus, we define true positive (TP) as the number of individuals whose estimated and true best treatments are both “treatment” (Z=1); true negative (TN) as the number of individuals whose treatment recommendation and true best treatment are both “control” (Z=0); false positive (FP) as the number of individuals whose estimated best treatment is “treatment” while the true best treatment is “control”; and false negative (FN) as the number of individuals whose estimated best treatment is “control” while the true best treatment is “treatment”. The following measurements were considered: accuracy $\left(\text{ACC}=\frac{\text{TP}+\text{TN}}{N}\right)$, positive predictive value $\left(\text{PPV}=\frac{\text{TP}}{\text{TP}+\text{FP}}\right)$, negative predictive value $\left(\text{NPV}=\frac{\text{TN}}{\text{TN}+\text{FN}}\right)$, sensitivity $\left(=\frac{\text{TP}}{\text{TP}+\text{FN}}\right)$, specificity $\left(=\frac{\text{TN}}{\text{TN}+\text{FP}}\right)$, and F-score $\left(=\frac{2}{{\text{PPV}}^{-1}+{\text{sensitivity}}^{-1}}\right)$.

### 3.3 Simulation Results

#### 3.3.1 Balanced Design

**Figure 1:**
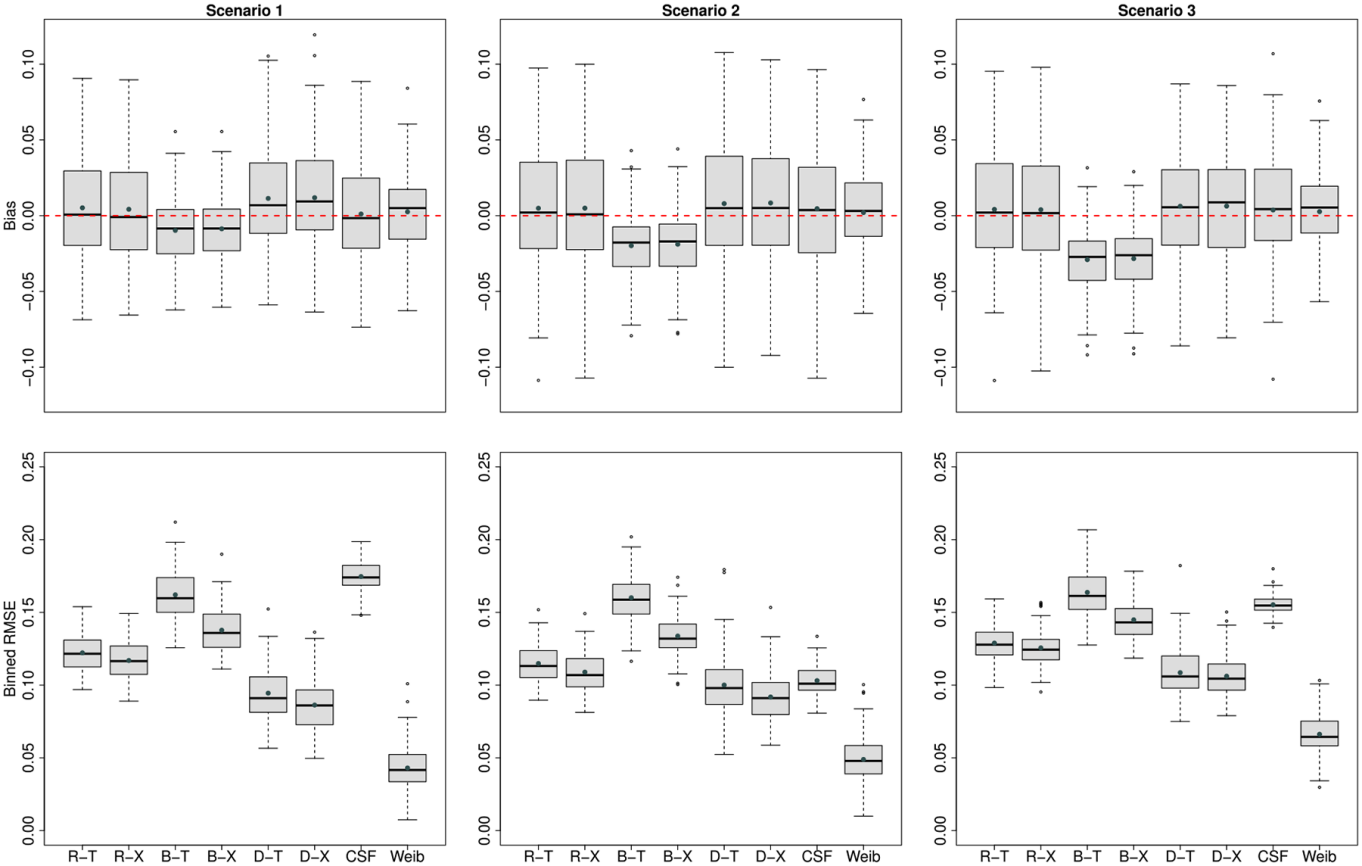
Simulation results when truths are generated from a **Weibull model**: box plots of biases (upper panel) and binned RMSEs (lower panel) to compare the performance of ITE estimates under the **balanced design** at the median failure time. The ITE estimates from the following combinations of the meta-algorithms and base learners were examined: RSF with T-learner (R-T), RSF with X-learner (R-X), BAFT with T-learner (B-T), BAFT with X-learner (B-X), DNNSurv with T-learner (D-T) and DNNSurv with X-learner (D-X). ‘CSF’ represents causal survival forests. ‘Weib’ represents the true Weibull model as the best case for ITE estimates.

Figure 1 presents the bias and binned RMSE for the ITE estimates from each method under the balanced design when the true survival time was simulated from the Weibull model. The ITE estimate from the correctly specified Weibull model demonstrates the best performance one can get. Generally, the methods with BAFT as the base learner (i.e., B-T and B-X) have relatively larger biases and RMSEs than other methods. The RSF-, DNNSurv-based methods and CSF have comparable biases under all scenarios, but DNNSurv-based methods provide smaller RMSEs in all three scenarios. The X-learner shows a slightly better performance in terms of RMSE than the T-learner with all three base learners. In general, CSF does not show better RMSE than the proposed methods. The prediction accuracy metrics are summarized in Table 1. Although CSF shows the best NPV and sensitivity across all three scenarios, it tends to predict more positive ITEs especially when scenarios get complicated (e.g. Scenarios 2 and 3) and yields low specificity. CSF predicts more than 90% positive ITEs while we only simulated 70% positive ITEs. Particularly, when scenarios get complicated, all predicted ITE values from CSF are positive in more than 50% runs of simulations. For the linear scenario (S1), X-learner with DNNSurv shows the best performance across all six metrics compared to the other five learners. For Scenarios 2 and 3, X-learner with DNNSurv demonstrates the best or the second-best performance for all metrics compared to the other five learners, except that X-learner with RSF has a higher sensitivity than DNNsurv-based learners in Scenario 3. Although R-X or D-T slightly outperforms D-X at some metrics under more complicated Scenarios 2 and 3, D-X provides the best prediction accuracy overall.

**Figure 2:**
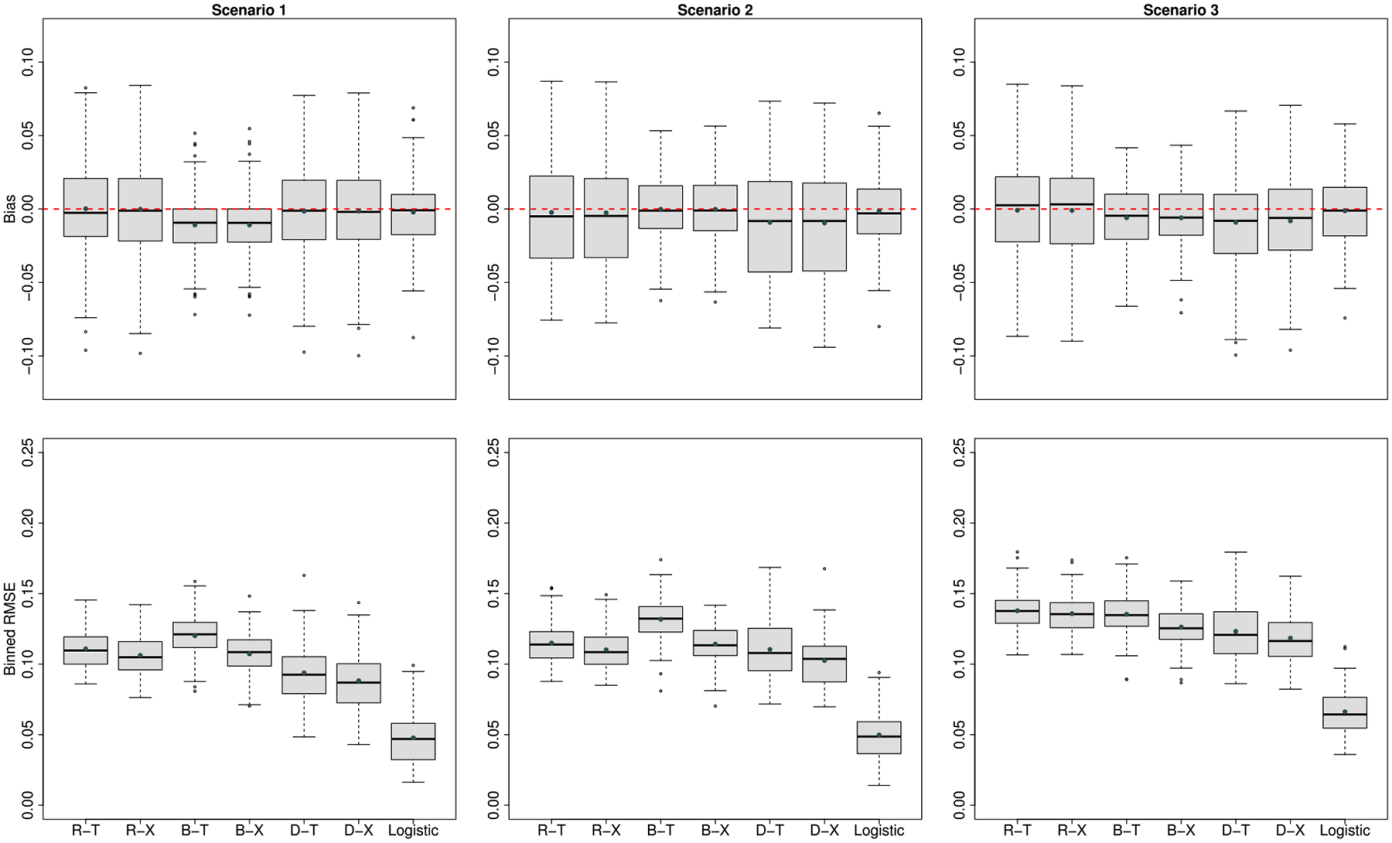
Simulation results when truths are generated from a **Log-logistic model**: box plots of bias (upper panel) and binned RMSE (lower panel) to compare the performance of ITE estimates under the **balanced design** at the median failure time. The abbreviation of the six proposed methods are the same as in Fig. 1. ‘Logistic’ represents the true Log-logistic model as the best case for ITE estimates.

Figure 2 presents the bias and binned RMSE for the ITE estimates from each method under the balanced design when the true survival time was simulated from the Log-logistic model. Although only BAFT satisfies the AFT assumption (the log-logistic model is an AFT model), RSF- and DNNSurv-based methods still perform well and even outperform BAFT in most cases. RSF-based methods show minimal biases in all three scenarios; BAFT-based methods show bias in Scenario 1, but the magnitudes are small; DNNSurv-based methods also show some small biases in Scenarios 2 and 3. For the binned RMSE, DNNSurv-based methods have smaller RMSEs than RSF- and BAFT-based methods in all three scenarios. The differences between T- and X-learners are small (except for BAFT methods), with X-learner showing smaller RMSEs in all three scenarios. Similar to Table 1, the prediction accuracy metrics under this Log-logistic setting are summarized in Table 2. D-X shows the best performance across all six metrics in all three scenarios.

Under both cases, although the propensity score was estimated, similar results were observed (not presented in the paper) when using the true propensity score for the weight function g(x)=0.5.

#### 3.3.2 Unbalanced Design

In the (very) unbalanced design in which the population overlapping assumption is slightly violated, with the original sample size for the training data being n=1,000 and the probability of getting treatment assignment being 0.05, the treatment arm only contains about 50 individuals, around 15 of them (30%) are censored. Such a small sample size can cause unstable results for machine-learning-based methods, especially the DNNSurv-based models. Based on our empirical experience with DNNSurv, it requires at least 100 events in each treatment arm to perform well. Therefore, we increased the sample size of the training dataset to n=4,000, which made the treatment arm contain about 200 individuals (~140 events). The test dataset still has a sample size of $N=10^{5}$.

Under the unbalanced design, the X-learner is expected to perform better than the T-learner since it accounts for the unbalanced allocation between the two treatment arms. BAFT with X-learner demonstrates an obvious advantage in RMSEs compared to BAFT with T-learner. In Scenarios 1 and 2, X-learner demonstrates smaller RMSE than T-learner under RSF- and DNNSur-based methods (Web Figure 1). RSF and DNNSurv with X-learner perform similarly to T-learner in Scenario 3. Overall, CSF does not show better performance in terms of RMSE, especially in Scenarios 1 and 3, but it performs similarly to the proposed methods in Scenario 2. Similar to the random design case, CSF predicts more than 90% positive ITE even though it shows the highest NPV and sensitivity. DNNSurv does not always demonstrate the best performer (Web Table 1). Moreover, DNNSurv produces larger RMSEs than RSF in complex scenarios (Scenarios 2 and 3), indicating that the neural network-based methods typically require a larger sample size to achieve stable performance as compared to other machine learning methods. Additional discussions are given in Section 5. Similar observations are found under the Log-logistic data case (results are not shown).

#### 3.3.3 Dependent Design

We further examine the performance under the case that confounders exist. The treatment assignment depends on covariates ($X_{1}$ and $X_{5}$) with data simulated from the Weibull model. The overall treatment allocation ratio was 1:1. The biases and RMSEs of the ITE estimates are presented in Web Figure 2, which are similar to the balanced case, indicating the robustness of the proposed methods when confounders exist. The DNNSurv-based methods still provide the best estimates with minimal biases and small RMSEs, followed by RSF-based methods. CSF still tends to predict more than 90% positive ITE values. Similar conclusions can be drawn from the prediction accuracy metrics (Web Table 2). DNNSurv-based methods have comparable results and tend to have the best or the second-best prediction accuracy in all six metrics.

Comparing across all scenarios and randomization designs, our simulations indicate that using DNNSurv to estimate base learners leads to more accurate ITE estimation in both T-learner and X-learner. Deep neural networks excel at capturing complex nonlinear trends, making them perform particularly well in complicated scenarios. Notably, DNNSurv under the X-learner framework (D-X) generally demonstrates the best performance in terms of accuracy for ITE estimates and treatment rule recommendations.

#### 3.3.4 Sensitivity Analysis

Since the ITE is defined at a pre-specified time t, we conducted sensitivity analysis with ITE being defined at a different time point (i.e., 25% and 75% percentile of survival times) under the Weibull case. Biases and binned RMSEs under each simulation design are shown in Web Figures 3, 4, and 5 for time = 25% percentile of survival times and in Web Figures 6, 7, and 8 for time = 75% percentile of survival times. In general, we reach the same conclusions compared to when median failure time was chosen as the time of interests. RSF- and DNNSurv-based methods show similar and minimal biases in all three randomization designs. DNNSurv-based methods show smaller RMSEs at both 25% and 75% percentile of survival times across all three scenarios under balanced and dependent designs. They have relatively larger RMSEs in scenarios 2 and 3 under the unbalanced design, but similar to the RMSEs of RSF-based methods and smaller than the RMSEs of BAFT-based methods. In all scenarios at these two different time points, X-learner shows smaller RMSEs than T-learner. CSF does not show better performance in terms of RMSEs and tends to predict more positive ITEs than the true positive ITEs.

#### 3.3.5 Computational Time

In this section, we compared the time required for running each of the six methods under scenario 1 of the balanced design as an illustration example, where the training sample size is 1,000 and test sample size is 10,000. The specific time costs for running each method once are listed in Table 3. In general, T-learner takes longer time compared to X-learner. This is because T-learner predicts ${\overset{ˆ}{S}}_{1}(t|x)$ and ${\overset{ˆ}{S}}_{0}(t|x)$ on the test data for each test sample (10,000 samples) before constructing the differences between ${\overset{ˆ}{S}}_{1}(t|x)$ and ${\overset{ˆ}{S}}_{0}(t|x)$. Instead, X-learner predicts ${\overset{ˆ}{S}}_{1}(t|x)$ and ${\overset{ˆ}{S}}_{0}(t|x)$ on each training sample (1,000 samples) to construct $\tilde{D}(t)$ in the first step. Thus, although X-learner has two steps, the gradient boosting in the second step is much faster than random survival forests in the first step. When using DNNSurv as base learners, T-learner and X-learner performs similarly. In terms of comparing different types of machine learning methods to estimate ${\overset{ˆ}{S}}_{1}(t|x)$ and ${\overset{ˆ}{S}}_{0}(t|x)$, RSF requires the least time comparing to BAFT and DNNSurv in both T-learner and X-learner. Although DNNSurv-based methods take relatively longer time, it is not computationally expensive.

## 4 Application to AREDS Study

### 4.1 Data Description

In this study, we analyzed a total of 806 participants with AMD (i.e., patients with AREDS AMD categories 2, 3, or 4) who were free of late-AMD in at least one eye at enrollment. The study included 466 (57.82%) female participants; around half of the participants (56.70%) had moderate AMD; 393 (48.76%) participants never smoked; the mean age of the participants was 68.77 (SD = 5.05), and the mean baseline AMD severity score was 4.09 (SD = 2.06). Participants were randomized to two treatment arms with 391 (48.51%) participants in the control (placebo) group and 415 (51.49%) participants in the AREDS formula group, and they did not differ in baseline age, sex, smoking status, and baseline AMD severity score between the two treatment arms. In our analysis, in addition to demographic and clinical variables, we also included 686 SNPs as covariates. These SNPs contain 46 SNPs identified to be associated with treatment efficacy (in delaying AMD progression) from Wei et al. (2021) and 640 SNPs identified to be associated with AMD progression (Yan et al., 2018) (with $p<10^{-5}$).

The standardized mean difference (SMD) is the most commonly used statistic to examine the balance of covariates between two treatment groups, where an SMD value of < 0.25 is considered indicative of balance (Belitser et al., 2011). In our study, SMD of all SNPs and demographic covariates are balanced between the treatment group and control group. All of the adjusted p-values from Fisher’s exact tests of SNPs versus treatment are greater than 0.05. Web Table 3 provides detailed summaries of participant baseline characteristics.

We use year five as our primary time of interest in estimating the ITE (i.e., progression-free probability at year five). We also performed a sensitivity analysis for estimating the ITE at year three.

### 4.2 Estimate ITE

**Figure 3:**
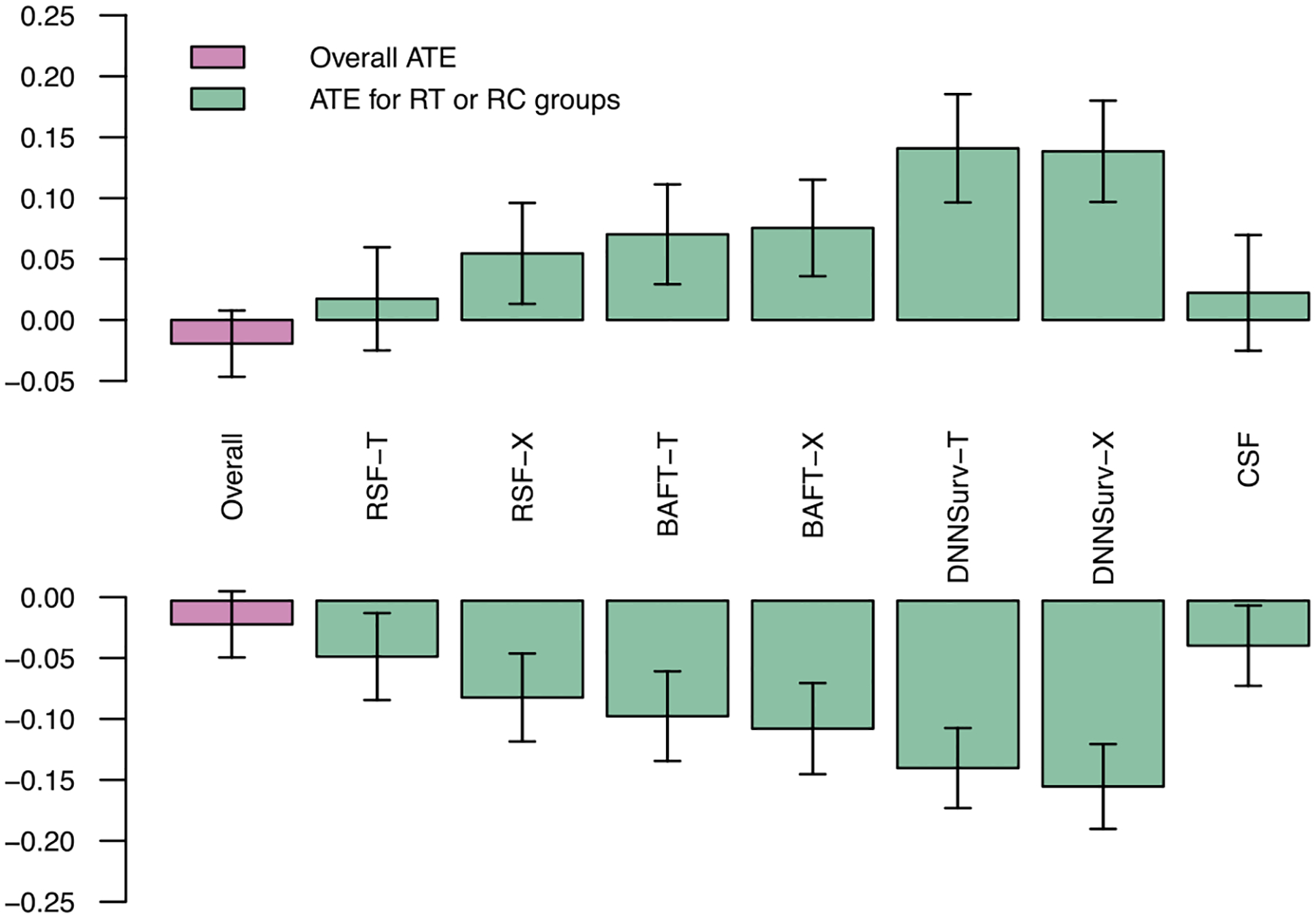
The mean treatment effect (KM estimates) at year five of participants in the RT cohort (recommended for taking the treatment) (upper panel) and in the RC cohort (recommended for taking the control/placebo) (lower panel) from each method in one random split of data.

Like the simulation studies, we applied the following proposed methods to estimate ITE: R-T, R-X, B-T, B-X, D-T, and D-X. We also compared these proposed methods with CSF. We considered three random splits of data stratified on treatment. For each split, 4-fold cross-validation was used to estimate ITE. For each method, participants with ITE estimate > 0 were labeled as RT (recommended for taking AREDS formula); otherwise, they were labeled as RC (recommended for taking control/placebo). Then within each cohort (RT or RC) recommended by each method, we estimated the mean treatment effect by taking the difference of Kaplan-Meier estimators (for the progression-free probabilities at five years) between the two treatment arms. Zhao et al. (2013) proposed a similar metric for selecting a target subgroup. We also estimated the overall treatment effect in the entire population using the same approach. Figure 3 shows the result for one random split of data and similar results are observed under two other random splits of data (Web Figure 7). From Figure 3, positive treatment effects are observed in the RT cohort under recommendations from all methods. Notably, participants in the RT cohort show the largest mean treatment effect under the recommendations based on D-T and D-X. Similarly, participants in the RC cohort also show the largest absolute mean treatment effect under the recommendations based on D-T and D-X. Note that the treatment effect in the RC cohort is expected to be negative since these participants should benefit from taking the placebo than the AREDS formula. CSF shows the smallest mean treatment effect across all three splits in both RT and RC cohorts. Note that the overall treatment effect (in all participants) is around 0, which is also consistent with what has been reported in the literature.

We performed the same analysis at year three. Web Figure 8 shows the mean treatment effect in RT and RC groups at year three for each method in three splits of data. Although D-X does not always perform the best, D-T and D-X perform similarly and show the largest mean treatment effects. CSF still shows the smallest mean treatment effects across three splits of data.

Our analysis demonstrates that D-T and D-X perform the best among all methods we applied. These two methods recommend about 40% to 50% participants for the AREDS formula.

### 4.3 Identify Important Features

Since D-X performs better than D-T in terms of the absolute treatment effect in the RC cohort (at year five), we used ITE estimates from D-X to make treatment recommendations for participants. Then we applied the Boruta algorithm (within each data split) to identify important features associated with the treatment recommendations. Top features identified by the Boruta algorithm were extracted, 41, 22, and 22 features were identified from each split, respectively, and all are SNPs. Note that 37 SNPs (out of 41) identified in split one and all of the 22 SNPs identified in split two and split three were previously reported in Wei et al. (2021), where SNPs were analyzed one by one to infer and identify subgroups with enhanced treatment efficacy using a multiple-testing-based approach. There are 17 common SNPs (from three gene regions *SPOCK2, C19orf44-CALR3*, and *ESRRB-VASH1*, located on chromosome 10, 19, and 14, respectively) being identified in all three splits and they were all reported in Wei et al. (2021).

When using the progression-free probability at year three as the treatment efficacy, we identified a similar amount of SNPs in three splits of data, and all of them were reported in Wei et al. (2021).

Since the SNPs within the same gene region are highly correlated (i.e., in tight linkage disequilibrium), we selected one SNP from each gene region and generated genotype distributions for each RC and RT cohort (from split 1) separately, and presented the result in Table 4 (top panel). Note that the participants recommended for control are less likely to be homozygous with two minor alleles in all three SNPs (i.e., the prevalence of aa in the RC cohort is less than that in the overall population). In contrast, participants recommended for the AREDS formula are more likely to have one or two copies of the minor allele (i.e., the prevalence of Aa and aa in the RT cohort is more than that in the overall population). Therefore, patients carrying minor alleles of these SNPs are more responsive to the AREDS formula. The Chi-square tests for the differences in genotype distributions between RC and RT are significant for all three SNPs. Note that in the analysis of year three, we identified 15 important SNPs, in which 14 SNPs are in common with the results of year five.

### 4.4 Validation on AREDS2

We validated the treatment recommendations generated from analyzing AREDS based on D-X on an independent dataset AREDS2, which is another large RCT of AMD (Chew et al., 2012). The study contained four treatment arms with different formulations of supplements (no placebo arm). One formulation is the same as the AREDS formula (from the AREDS study). The participants in AREDS2 were more severe than those in AREDS. Therefore, to ensure a similar participant population as AREDS, we included 110 participants who were randomized to the AREDS formula and had mild-to-moderate AMD (baseline AMD severity score: 2 to 6). The baseline characteristics of these validation participants are similar the AREDS participants: mean baseline severity scale was 5.43 (SD = 0.96); mean age was 71.29 (SD = 7.25); 50 (44.64%) participants were females, and 44 (39.29%) participants never smoked.

We applied D-X 5 times to estimate ITE at year five. Participants with averaged estimated ITE > 0 (among five runs) were recommended for the AREDS formula (RT); otherwise, they were not recommended for the AREDS formula (RC). In summary, D-X recommends 45% participants for the AREDS formula. Web Figure 9 shows that the overall 5-year progression-free probability is significantly higher in the RT group than in the RC group (log-rank test p=0.011). The bottom panel of Table 4 shows genetic profiles of RT and RC groups from AREDS2. We found consistent results as in AREDS, i.e., participants who were not recommended for the AREDS formula are less likely to be homozygous with two copies of the minor allele for all three SNPs. In contrast, participants recommended for the AREDS formula are more likely to have one or two copies of the minor allele for all three SNPs. Fisher’s exact tests for the differences in genotype distributions between RC and RT are also significant for all three SNPs. We observed similar results at year three.

## 5 Conclusion and Discussion

In this work, we developed two meta-algorithms (T-learner and X-learner) on survival outcomes using RSF, BAFT, and DNNSurv as base learners to estimate ITE and to provide treatment rule recommendations. The performance of these methods has been evaluated through comprehensive simulations. We found that the DNNSurv-based learners generally outperform other methods in terms of biases and RMSEs in estimating ITEs. RSF-based methods have comparable biases with DNNSurv-based methods but larger RMSEs. As a comparison method, CSF shows the least favorable performance in all scenarios and tends to predict more positive ITE. X-learner demonstrates advantages in balancing sample sizes under the unbalanced treatment allocation setting.

We applied these meta-learners to AREDS data to identify individuals who could benefit from the AREDS formula. We found that the T- and X-learners with the DNNSurv model identified the group of participants with the greatest enhanced treatment effects. The Boruta algorithm was applied to identify important features contributing to the treatment recommendation rule. SNPs from gene regions reported in the previous study to be associated with differential treatment efficacy were successfully identified as important variables predicting ITEs in our study. The treatment recommendation rule was further validated on participants from an independent study AREDS2.

We also conducted a sensitivity analysis in both simulations and real data analysis in estimating ITEs at different time points. We reach to the same conclusions showing that our proposed meta-learners are relatively robust to different time points.

One limitation of the DNNSurv-based methods is that they require a sizable cohort to train multiple tuning hyperparameters. The performance could be unstable if the sample size is not large enough. We also observe that the X-learner does not demonstrate substantially improved performance over the T-learner, except for the unbalanced design with no average treatment effect. One plausible explanation is that we use the estimated survival probability for both factual and counterfactual parts in the first step of X-learner to compute the T-learner type of ITE, where the original X-learner for continuous or binary outcomes uses the observed outcome in the factual part, which is more trustful. Other imputation approaches, such as the inverse probability weighted method (e.g., based on the observed progression status for the factual part), may be considered for the survival outcome X-learner. Furthermore, we assumed non-informative censoring assumption and did not discuss the situation when this assumption is violated, as our focus in this paper is RCTs and this assumption typically holds in RCTs. We might model the censoring mechanism under the informative censoring case and extend the current approaches to incorporate informative censoring. Finally, in this paper, we did not perform sample splitting when performing X-learner (i.e., split the dataset into two subsets and use each dataset for each step) since the performance has been observed similar between splitting the data or not. This has also been demonstrated in Curth and van der Schaar (2021).

## Supplementary Material

HTE_Supp_JDS

## Figures and Tables

**Table 1:** Simulation results when truths are generated from a **Weibull model**: prediction accuracy under the **balanced design** at the median failure time. Six metrics are summarized with mean (SD) for each method under each scenario: overall accuracy (ACC), positive predictive value (PPV), negative predictive value (NPV), sensitivity, specificity, and F-score.

	R-T	R-X	B-T	B-X	D-T	D-X	CSF	Weib
Scenario 1								
ACC	90.11 (1.81)	90.58 (1.85)	85.99 (2.03)	88.55 (1.91)	92.39 (1.71)	93.44 (1.67)	82.02 (7.15)	96.37 (1.62)
PPV	91.78 (3.21)	92.33 (3.30)	90.67 (2.32)	92.18 (2.50)	94.96 (3.02)	95.52 (2.94)	81.18 (7.56)	97.36 (2.33)
NPV	87.17 (5.99)	87.56 (6.19)	76.13 (4.54)	80.93 (4.81)	87.62 (5.77)	89.63 (5.43)	95.55 (6.05)	94.68 (4.90)
Sensitivity	94.56 (3.24)	94.62 (3.38)	89.26 (2.96)	91.51 (2.94)	94.30 (3.25)	95.25 (3.04)	98.38 (2.67)	97.55 (2.44)
Specificity	79.74 (9.21)	81.16 (9.29)	78.36 (6.22)	81.64 (6.66)	87.92 (8.00)	89.20 (7.63)	43.86 (27.72)	93.62 (5.89)
F-score	93.05 (1.22)	93.36 (1.27)	89.90 (1.52)	91.79 (1.39)	94.54 (1.22)	95.31 (1.20)	88.67 (3.84)	97.41 (1.15)
Scenario 2								
ACC	79.68 (4.01)	80.50 (4.15)	72.15 (3.78)	75.29 (3.91)	82.10 (4.50)	82.76 (4.68)	76.31 (5.49)	91.04 (3.53)
PPV	86.55 (4.87)	86.97 (5.11)	84.69 (3.50)	86.18 (4.01)	89.60 (5.28)	89.63 (5.78)	78.84 (8.21)	94.07 (4.47)
NPV	66.96 (9.64)	68.80 (10.19)	51.73 (5.27)	56.37 (6.08)	70.15 (11.31)	72.14 (11.67)	82.95 (14.92)	86.84 (10.55)
Sensitivity	85.31 (8.21)	86.15 (8.26)	74.42 (5.21)	78.00 (5.61)	85.38 (8.42)	86.57 (8.68)	94.02 (9.68)	93.69 (6.01)
Specificity	65.89 (16.12)	66.65 (16.80)	66.60 (9.65)	68.66 (11.45)	74.07 (16.28)	73.41 (18.48)	32.90 (31.65)	84.53 (12.63)
F-score	85.50 (3.47)	86.12 (3.51)	79.08 (3.20)	81.70 (3.19)	87.00 (3.74)	87.57 (3.78)	84.89 (4.15)	93.64 (2.65)
Scenario 3								
ACC	83.47 (2.16)	83.74 (2.20)	79.21 (2.83)	81.30 (2.57)	86.73 (2.58)	86.77 (2.19)	74.33 (3.36)	92.12 (1.89)
PPV	86.61 (3.66)	86.42 (3.86)	87.52 (2.46)	88.06 (2.57)	91.22 (3.19)	90.34 (3.70)	74.18 (3.96)	94.86 (2.53)
NPV	76.62 (7.85)	78.17 (7.93)	62.53 (5.16)	66.87 (5.52)	77.89 (8.26)	79.66 (7.75)	94.78 (8.29)	86.67 (6.79)
Sensitivity	91.33 (4.92)	92.08 (4.77)	82.76 (4.15)	85.53 (4.31)	90.35 (5.21)	91.58 (4.73)	98.97 (3.09)	94.20 (3.63)
Specificity	63.91 (12.66)	62.95 (13.52)	70.37 (7.06)	70.77 (7.78)	77.73 (9.68)	74.77 (11.60)	13.00 (17.12)	86.96 (7.14)
F-score	88.72 (1.59)	88.97 (1.50)	84.99 (2.28)	86.67 (2.06)	90.62 (2.05)	90.78 (1.60)	84.66 (1.57)	94.45 (1.41)

**Table 2:** Simulation results when truths are generated from a **Log-logistic model**: prediction accuracy under **balanced design** at the median failure time. Abbreviations of the metrics and methods are the same as in Table 1.

	R-T	R-X	B-T	B-X	D-T	D-X	Logistic
Scenario 1							
ACC	79.04 (4.40)	79.75 (4.53)	78.63 (3.65)	80.65 (3.73)	83.23 (4.83)	83.75 (5.06)	91.56 (4.28)
PPV	84.06 (5.25)	84.67 (5.51)	85.21 (4.18)	86.58 (4.57)	87.62 (5.84)	87.72 (5.81)	94.32 (5.20)
NPV	72.20 (7.75)	73.24 (7.91)	69.28 (5.86)	72.34 (6.28)	78.02 (8.68)	79.26 (9.47)	88.74 (8.56)
Sensitivity	83.84 (7.56)	84.38 (7.68)	81.03 (5.81)	83.07 (5.98)	86.81 (7.58)	87.59 (7.71)	92.87 (6.22)
Specificity	70.49 (12.90)	71.51 (13.37)	74.34 (9.16)	76.33 (9.99)	76.85 (13.63)	76.91 (13.55)	89.22 (10.73)
F-score	83.58 (3.85)	84.13 (3.94)	82.87 (3.18)	84.56 (3.18)	86.83 (4.03)	87.28 (4.16)	93.34 (3.44)
Scenario 2							
ACC	80.98(3.54)	82.05 (3.67)	79.21 (3.18)	81.97 (3.20)	83.13 (4.29)	83.99 (4.20)	91.90 (3.73)
PPV	81.45 (6.34)	82.54 (6.59)	78.84 (4.39)	81.66 (4.81)	83.31 (6.89)	84.34 (6.95)	92.68 (6.23)
NPV	82.19 (6.00)	83.39 (6.36)	80.12 (4.06)	82.99 (4.39)	84.71 (6.63)	85.70 (7.26)	92.41 (5.94)
Sensitivity	81.13 (8.74)	82.28 (9.04)	79.80 (5.53)	82.61 (5.83)	83.79 (9.05)	84.53 (9.83)	91.58 (7.47)
Specificity	80.84 (9.18)	81.83 (9.36)	78.64 (5.97)	81.35 (6.40)	82.48 (9.48)	83.46 (9.58)	92.21 (7.25)
F-score	80.74 (3.92)	81.83 (4.07)	79.13 (3.27)	81.91 (3.25)	83.00 (4.59)	83.79 (4.68)	91.75 (3.98)
Scenario 3							
ACC	82.12 (2.28)	82.15 (2.35)	83.39 (2.39)	84.16 (2.33)	85.63 (2.72)	85.68 (2.60)	92.60 (2.00)
PPV	83.61 (4.36)	83.48 (4.50)	85.86 (3.16)	86.11 (3.35)	86.64 (4.23)	86.76 (4.54)	93.98 (2.25)
NPV	81.37 (5.63)	81.83 (6.00)	80.45 (3.99)	82.00 (4.24)	85.25 (5.64)	85.37 (5.57)	91.26 (4.12)
Sensitivity	86.78 (6.08)	87.12 (6.41)	85.69 (4.05)	86.99 (4.29)	89.47 (5.24)	89.49 (5.36)	93.43 (3.65)
Specificity	75.66 (8.82)	75.26 (9.26)	80.19 (5.46)	80.22 (5.93)	80.32 (7.73)	80.40 (8.34)	91.44 (5.10)
F-score	84.89 (2.12)	84.95 (2.20)	85.68 (2.16)	86.42 (2.10)	87.83 (2.36)	87.87 (2.22)	93.61 (1.76)

**Table 3:** Computational time for each method. Each method was run once under scenario 1 of the balanced design, with training sample size 1,000 and test sample size 10,000. Time costs of D-T and D-X were tracked through Python (Tensorflow1). Other methods were run in Rstudio.

Method	Computational time (seconds)
R-T	25.68
R-X	5.80
B-T	409.68
B-X	79.46
D-T	398.61
D-X	407.10

**Table 4:** Genotype distributions in the overall and each treatment recommendation group (RT and RC) in two studies (AREDS and AREDS2) when time of interest is year five in estimating ITE.

AREDS					
	Genotype	Overall (*n* = 806)	RC (*n* = 436)	RT (*n* = 370)	p-value*
rs1245576 (*SPOCK2*, CHR10)	AA	266 (33.00%)	199 (45.64%)	67 (18.11%)	<. 0001
	Aa	408 (50.62%)	205 (47.02%)	203 (54.86%)	
	aa	132 (16.38%)	32 (7.34%)	100 (27.03%)	
rs8109218 (*C19orf44-CALR3*, CHR19)	AA	388 (48.14%)	254 (58.26%)	134 (36.22%)	<. 0001
	Aa	333 (41.32%)	160 (36.70%)	173 (46.76%)	
	aa	85 (10.55%)	22 (5.05%)	63 (17.03%)	
rs147106198 (*ESRRB-VASH1*, CHR14)	AA	387 (48.01%)	249 (57.11%)	138 (37.30%)	<. 0001
	Aa	343 (42.56%)	168 (38.53%)	175 (47.30%)	
	aa	76 (9.43%)	19 (4.36%)	57 (15.41%)	
AREDS2					
	Genotype	Overall (*n* = 110)	RC (*n* = 60)	RT (*n* = 50)	p-value*
rs1245576 (*SPOCK2*, CHR10)	AA	37 (33.64%)	29 (48.33%)	8 (16.00%)	<. 0001
	Aa	57 (51.82%)	29 (48.33%)	28 (56.00%)	
	aa	16 (14.55%)	2 (3.33%)	14 (28.00%)	
rs8109218 (*C19orf44-CALR3*, CHR19)	AA	44 (40.00%)	33 (55.00%)	11 (22.00%)	0.0018
	Aa	54 (49.09%)	23 (38.33%)	31 (61.00%)	
	aa	12 (10.91%)	4 (6.67%)	8 (16.00%)	
rs147106198 (*ESRRB-VASH1*, CHR14)	AA	49 (44.55%)	38 (63.33%)	11 (22.00%)	<. 0001
	Aa	53 (48.18%)	19 (31.67%)	34 (68.00%)	
	aa	8 (7.27%)	3 (5.00%)	5 (10.00%)	

* Chi-squared or Fisher’s exact tests were performed to examine the differences between genotype groups and treatment recommendation groups.
