# Supplementary material for: A Meta-Learner Framework to Estimate Individualized Treatment Effects for Survival Outcomes
Source: J Data Sci. Author manuscript; Available in PMC 2025 Sep 17. (PMC12440118; doi:10.6339/24-jds1119)
Supplement: HTE_Supp_JDS [file NIHMS2072922-supplement-HTE_Supp_JDS.pdf]

Supporting Information for
“A Meta-Learner Framework to Estimate Individualized Treatment Effects for Survival Outcomes”

by Na Bo, Yue Wei, Lang Zeng, Chaeryon Kang, and Ying Ding

Contents

1 Appendix S1: Additional figures and tables from simulations 1
- 1.1 Unbalanced design 1
- 1.2 Dependent design 1
- 1.3 Sensitivity analysis 5

2 Appendix S2: Additional figures and tables for real data analysis 11
- 2.1 AREDS data analysis 11
- 2.2 AREDS2 validation data analysis 11

1 Appendix S1: Additional figures and tables from simulations

1.1 Unbalanced design

Web Figure 1 shows the box plots of biases and binned RMSEs and Web Table 1 shows the prediction accuracy of 100 simulations runs with a sample size of 4000 in the training dataset for the unbalanced design ($P(Z = 1) = 0.05$) at the median failure time where data were simulated from a Weibull distribution with nonzero treatment effect.

1.2 Dependent design

Web Figure 2 shows the box plots of biases and binned RMSEs at the median failure time to compare the performance of ITE estimates under the dependent design (the probability of treatment assignment depends on covariates X_1 and X_5). Web Table 2 presents the corresponding prediction accuracy.

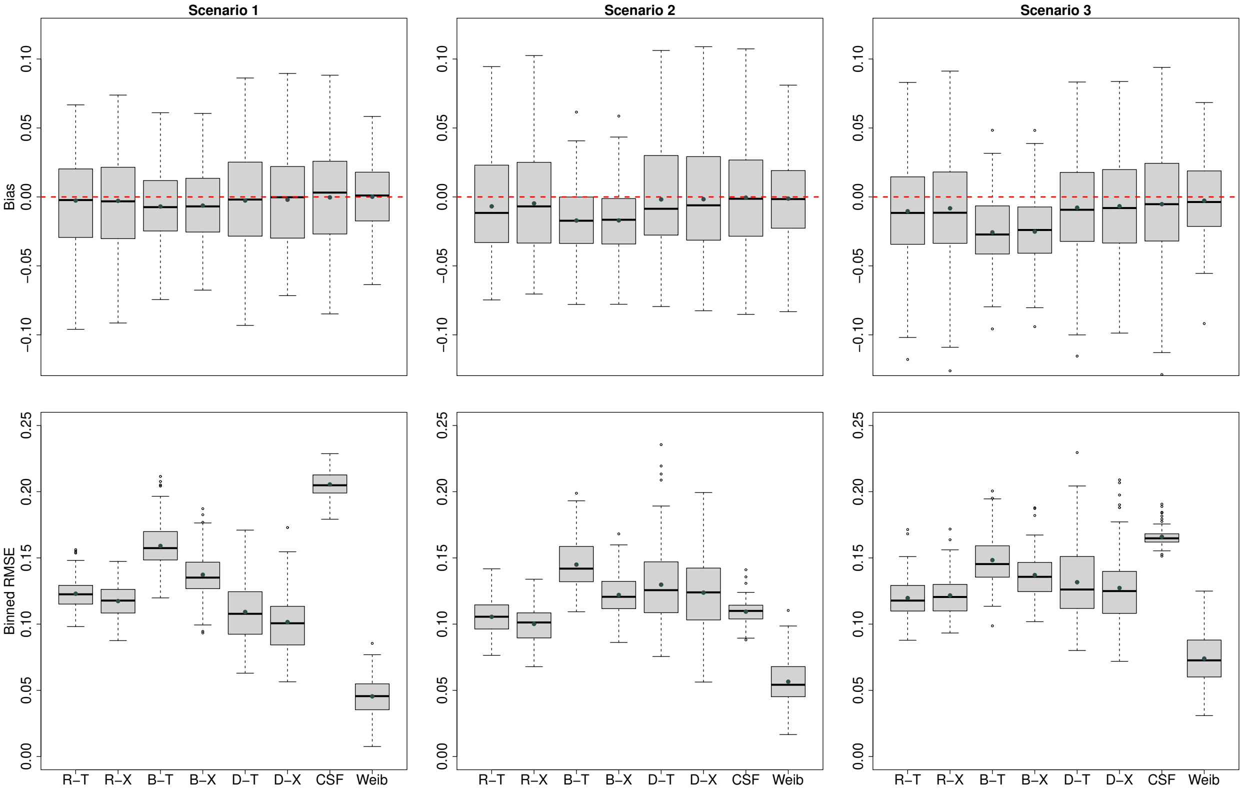

Web Figure 1: Simulation results when truths are generated from a **Weibull model**: box plots of biases (upper panel) and binned RMSEs (lower panel) to compare the performance of ITE estimates under **unbalanced design** at the median failure time with sample size (of the training dataset) as 4000. The ITE estimates from the following combinations of the meta-algorithms and base learners were examined: RSF with T-learner (R-T), RSF with X-learner (R-X), BAFT with T-learner (B-T), BAFT with X-learner (B-X), DNNSurv with T-learner (D-T) and DNNSurv with X-learner (D-X). 'CSF' represents the causal survival forest method, and 'Weib' represents the true Weibull model as the best case for ITE estimates.

Web Table 1: Simulation results when truths are generated from a **Weibull model**: prediction accuracy under **unbalanced design** at the median failure time with sample size as 4000. Six metrics are summarized with mean (SD) for each method under each scenario: overall accuracy (ACC), positive predictive value (PPV), negative predictive value (NPV), sensitivity, specificity and F-score.

	R-T	R-X	B-T	B-X	D-T	D-X	CSF	Weibull
				Scenario 1				
ACC	89.87 (2.10)	90.40 (2.08)	86.38 (2.38)	88.98 (2.11)	90.27 (2.16)	91.18 (2.03)	79.24 (7.85)	95.75 (1.80)
PPV	92.06 (3.74)	92.63 (3.88)	90.45 (2.29)	92.02 (2.56)	94.10 (3.01)	94.64 (2.88)	79.07 (9.05)	97.12 (2.35)
NPV	86.33 (7.06)	86.96 (7.15)	77.53 (5.32)	82.53 (5.51)	83.14 (6.62)	84.75 (6.50)	95.58 (7.39)	93.28 (5.45)
Sensitivity	93.94 (4.22)	94.10 (4.37)	90.14 (3.12)	92.38 (3.14)	92.05 (4.00)	92.82 (3.84)	98.17 (3.71)	96.89 (2.77)
Specificity	80.39 (10.46)	81.77 (10.71)	77.62 (6.11)	81.05 (6.80)	86.11 (7.80)	87.34 (7.50)	35.11 (32.37)	93.09 (5.88)
F-score	92.84 (1.50)	93.20 (1.52)	90.25 (1.76)	92.14 (1.53)	92.95 (1.65)	93.62 (1.54)	87.15 (4.15)	96.95 (1.31)
				Scenario 2				
ACC	80.06 (3.85)	80.48 (3.87)	74.88 (4.69)	77.42 (4.91)	78.53 (5.64)	78.56 (5.48)	75.16 (6.53)	89.22 (4.67)
PPV	89.58 (4.22)	89.31 (4.70)	86.55 (3.65)	87.69 (4.23)	87.91 (5.39)	87.86 (5.53)	77.79 (7.25)	93.91 (4.25)
NPV	64.71 (8.55)	65.90 (8.61)	55.80 (6.91)	59.82 (7.94)	62.53 (10.51)	62.75 (10.41)	79.54 (15.50)	82.25 (12.17)
Sensitivity	81.92 (7.77)	83.03 (7.93)	76.69 (6.27)	79.62 (6.63)	81.50 (8.25)	81.69 (8.41)	93.67 (12.86)	91.11 (7.76)
Specificity	75.52 (12.66)	74.23 (14.43)	70.43 (9.43)	72.01 (11.39)	71.25 (15.59)	70.90 (16.24)	29.80 (29.66)	84.57 (11.88)
F-score	85.22 (3.45)	85.66 (3.41)	81.16 (3.98)	83.26 (4.03)	84.21 (4.56)	84.26 (4.47)	83.88 (7.12)	92.18 (3.81)
				Scenario 3				
ACC	84.41 (2.64)	84.04 (2.68)	81.82 (2.87)	82.94 (2.72)	84.31 (3.80)	84.61 (3.70)	74.16 (3.75)	91.15 (2.65)
PPV	90.01 (3.57)	88.54 (3.97)	89.50 (2.56)	88.83 (2.70)	90.93 (3.80)	90.73 (3.82)	76.97 (6.95)	94.99 (2.77)
NPV	73.36 (7.49)	75.03 (8.27)	66.58 (5.58)	68.99 (6.14)	71.65 (8.16)	72.78 (8.76)	84.06 (19.55)	83.68 (7.73)
Sensitivity	**88.28 (5.60)**	89.66 (5.91)	84.55 (4.10)	86.47 (4.37)	86.95 (5.75)	87.66 (5.77)	93.61 (10.81)	92.62 (4.38)
Specificity	74.77 (11.24)	70.05 (13.17)	75.03 (7.26)	72.56 (8.19)	77.74 (10.83)	77.01 (11.07)	25.74 (29.93)	87.47 (7.47)
F-score	88.93 (2.19)	88.85 (2.14)	86.87 (2.25)	87.53 (2.13)	88.71 (2.97)	88.98 (2.86)	83.65 (3.03)	93.69 (2.02)

Web Table 2: Simulation results when truths are generated from a **Weibull model**: prediction accuracy under the **dependent design** at the median failure time.

	R-T	R-X	B-T	B-X	D-T	D-X	CSF	Weibull
				Scenario 1				
ACC	89.86 (1.67)	90.41 (1.72)	85.64 (2.18)	88.21 (1.94)	92.23 (2.34)	92.99 (2.25)	81.54 (6.55)	96.29 (1.88)
PPV	91.47 (3.07)	92.10 (3.17)	90.78 (1.95)	92.34 (2.16)	95.25 (2.38)	95.99 (2.26)	80.66 (7.35)	97.71 (2.15)
NPV	87.17 (6.46)	87.68 (6.65)	75.12 (5.09)	79.78 (5.28)	86.68 (6.90)	87.56 (6.88)	95.93 (6.34)	93.70 (5.39)
Sensitivity	94.54 (3.64)	94.65 (3.75)	88.53 (3.38)	90.78 (3.31)	93.70 (4.28)	94.03 (4.24)	98.45 (3.00)	97.06 (2.78)
Specificity	78.94 (8.77)	80.55 (8.90)	78.88 (5.23)	82.23 (5.76)	88.80 (6.03)	90.55 (5.72)	42.11 (26.37)	94.52 (5.37)
F-score	92.87 (1.21)	93.24 (1.25)	89.59 (1.71)	91.49 (1.50)	94.37 (1.89)	94.90 (1.82)	88.38 (3.50)	97.34 (1.38)
				Scenario 2				
ACC	79.03 (4.68)	79.71 (4.88)	71.93 (4.35)	74.76 (4.55)	81.79 (5.62)	82.24 (5.27)	74.71 (5.68)	90.45 (4.82)
PPV	86.59 (4.76)	86.97 (5.00)	84.66 (3.60)	86.11 (4.19)	90.64 (5.18)	90.56 (5.33)	77.41 (7.72)	94.37 (4.80)
NPV	64.76 (9.15)	66.18 (9.46)	51.51 (5.89)	55.52 (6.62)	68.61 (11.08)	69.38 (10.42)	82.58 (16.74)	84.63 (11.40)
Sensitivity	84.06 (8.20)	84.74 (8.48)	74.03 (5.63)	77.16 (5.95)	83.65 (10.09)	84.50 (9.45)	93.98 (12.25)	92.51 (7.18)
Specificity	66.72 (15.36)	67.38 (16.19)	66.79 (9.37)	68.89 (11.39)	77.21 (15.06)	76.70 (16.01)	27.50 (29.71)	85.42 (13.33)
F-score	84.91 (4.08)	85.41 (4.29)	78.85 (3.72)	81.20 (3.75)	86.42 (5.48)	86.88 (5.07)	83.76 (6.03)	93.14 (3.76)
				Scenario 3				
ACC	83.68 (2.26)	83.98 (2.29)	79.55 (2.84)	81.49 (2.68)	86.94 (2.81)	86.83 (2.39)	74.29 (3.57)	91.94 (2.33)
PPV	86.71 (3.52)	86.62 (3.72)	87.94 (2.74)	88.44 (2.99)	91.85 (3.64)	91.09 (3.77)	74.23 (4.40)	95.36 (2.97)
NPV	76.81 (7.44)	78.42 (7.88)	62.95 (5.13)	66.93 (5.49)	77.71 (8.41)	78.71 (8.11)	95.58 (8.45)	85.25 (6.87)
Sensitivity	91.48 (4.82)	92.16 (4.88)	82.83 (4.17)	85.40 (4.28)	90.00 (5.38)	90.77 (5.11)	98.89 (3.24)	93.42 (3.91)
Specificity	64.24 (12.09)	63.61 (12.88)	71.38 (7.99)	71.76 (9.04)	79.34 (11.18)	77.02 (11.80)	13.04 (18.90)	88.25 (8.20)
F-score	88.86 (1.66)	89.12 (1.63)	85.21 (2.26)	86.78 (2.08)	90.72 (2.18)	90.74 (1.83)	84.64 (1.64)	94.28 (1.74)

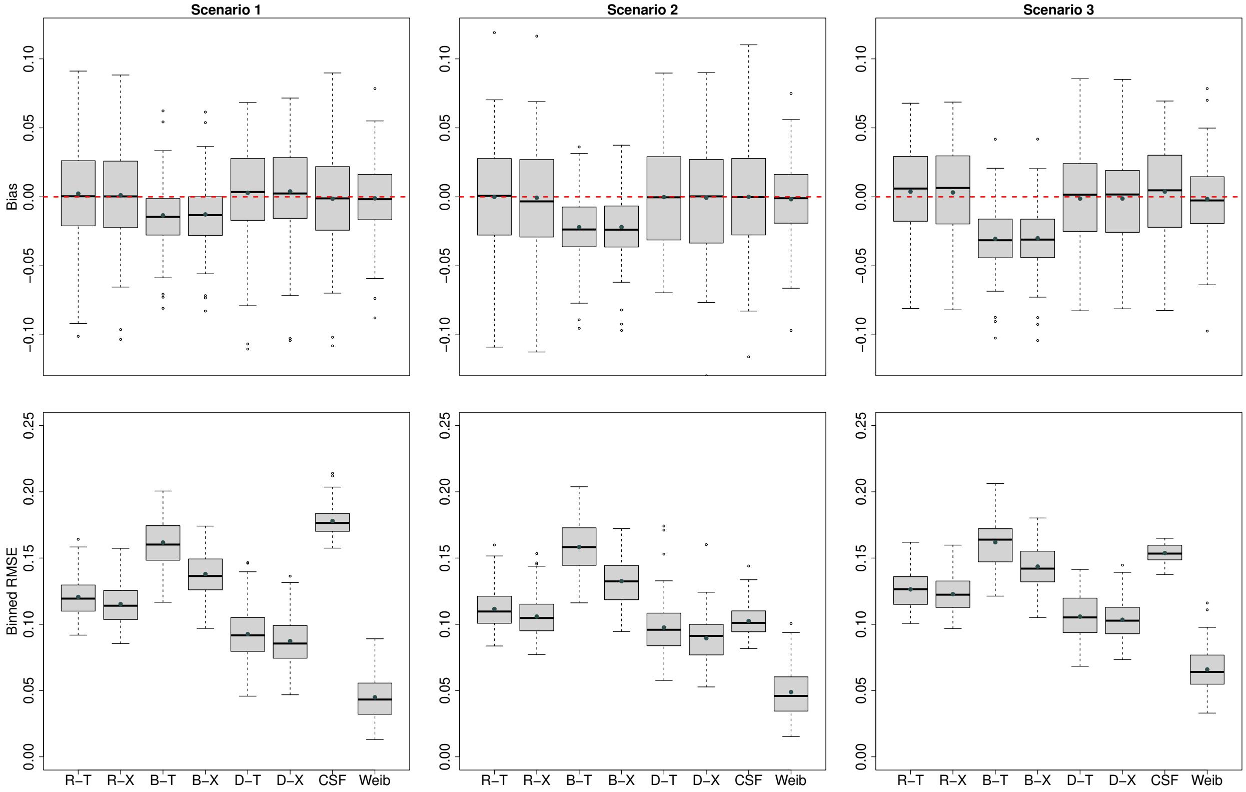

Web Figure 2: Simulation results when truths are generated from a **Weibull model**: box plots of biases and binned RMSEs to compare the performance of ITE estimates under **dependent design** at the median failure time.

1.3 Sensitivity analysis

Web Figures 3, 4, and 5 show the box plots of biases and binned RMSEs to compare the performance of ITE under balanced, dependent and unbalanced design at the 25% percentile of survival times, respectively.

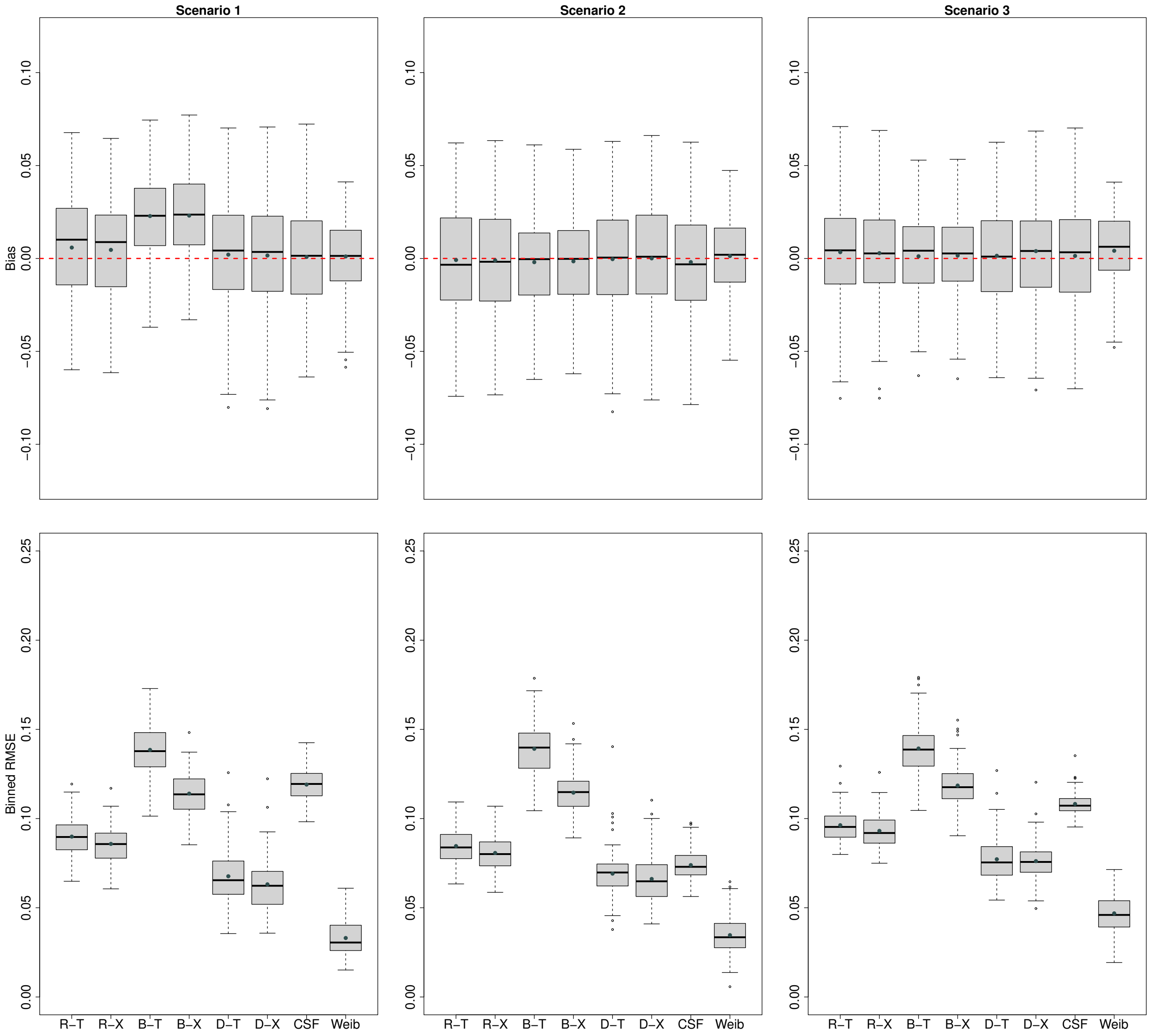

Web Figure 3: Simulation results at the **25% percentile of survival times** when truths are generated from a **Weibull model**: box plots of biases (upper panel) and binned RMSEs (lower panel) to compare the performance of ITE estimates under **balanced design**.

Web Figures 6, 7, and 8 show the box plots of biases and binned RMSEs to compare the performance of ITE under balanced, dependent and unbalanced design at the 75% quantile of survival times, respectively.

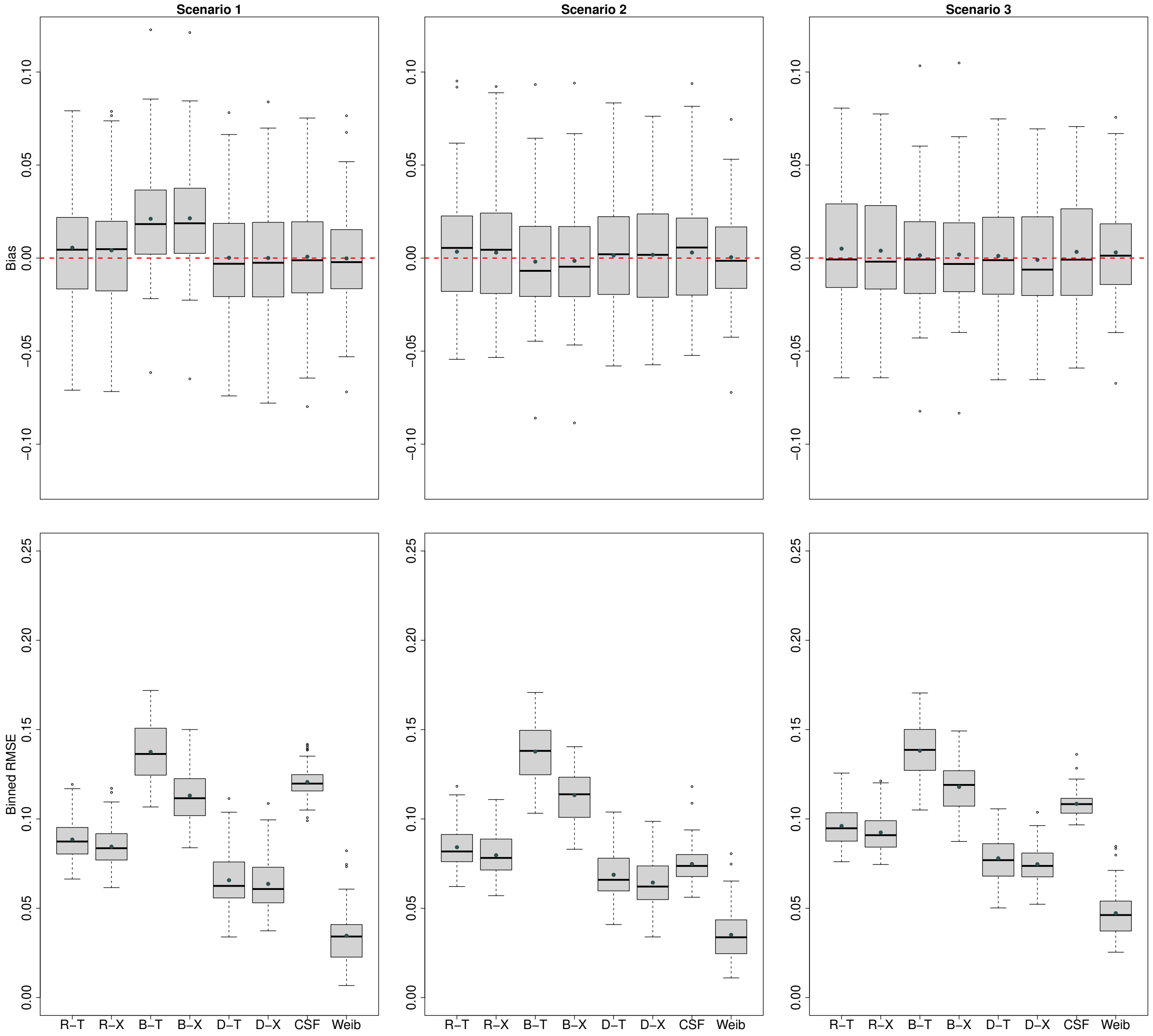

Web Figure 4: Simulation results at the **25% percentile of survival times** when truths are generated from a **Weibull model**: box plots of biases (upper panel) and binned RMSEs (lower panel) to compare the performance of ITE estimates under **dependent design**.

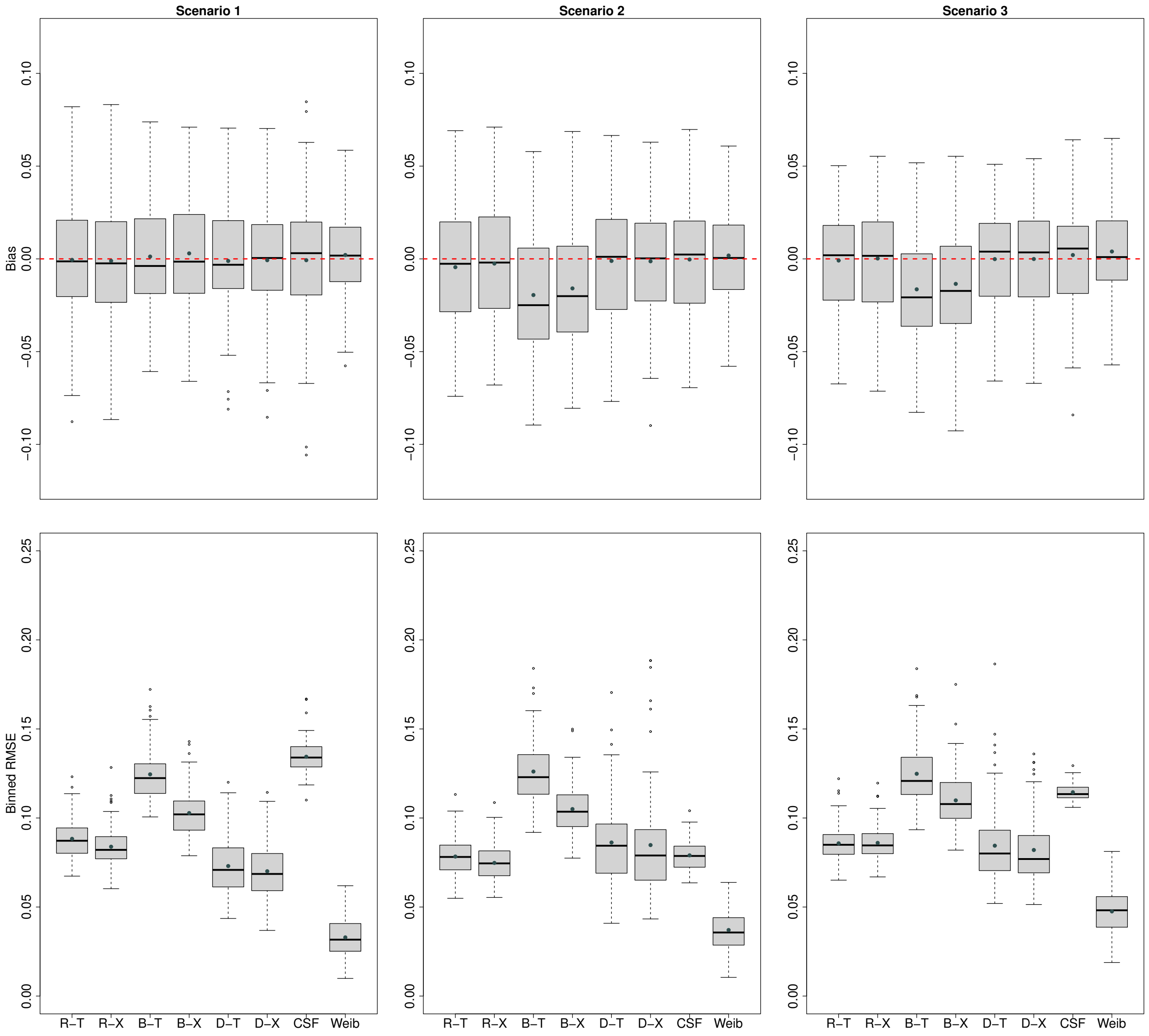

Web Figure 5: Simulation results at the **25% percentile of survival times** when truths are generated from a **Weibull model**: box plots of biases (upper panel) and binned RMSEs (lower panel) to compare the performance of ITE estimates under **unbalanced design** with sample size of 4000.

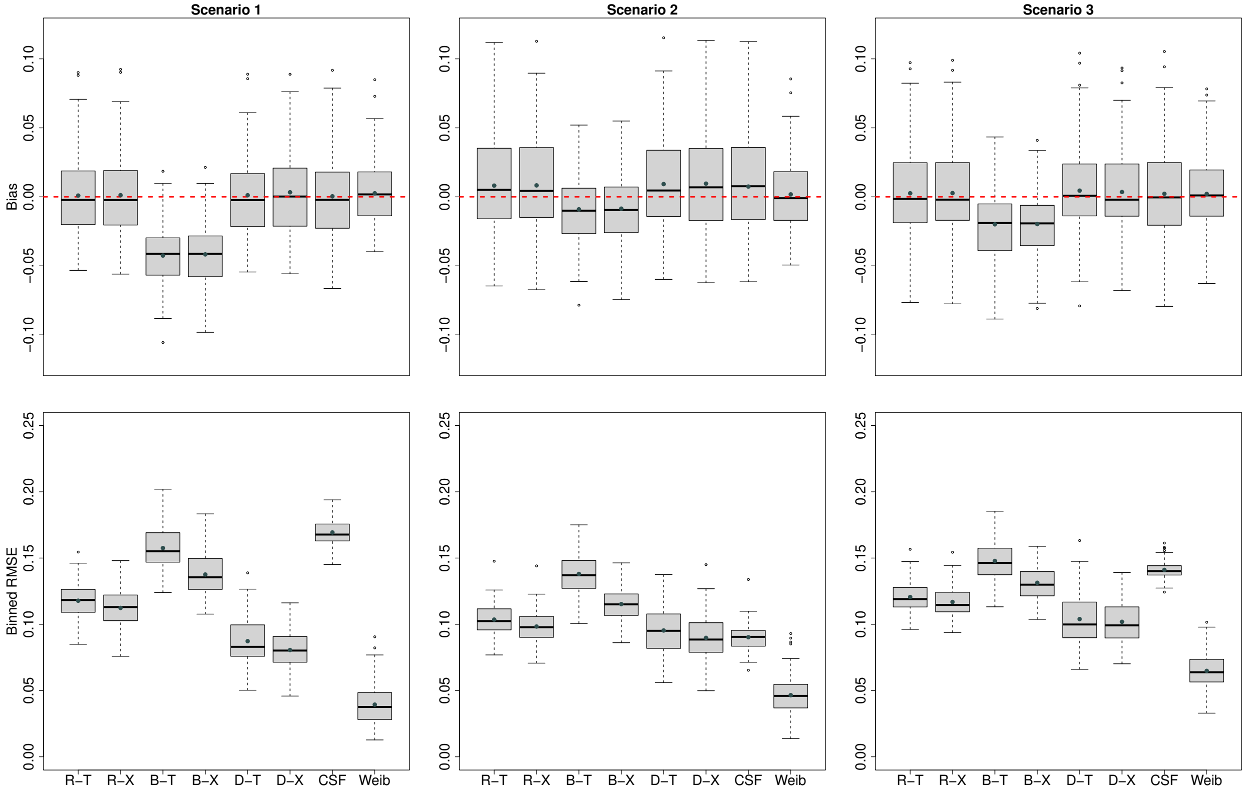

Web Figure 6: Simulation results at the **75% percentile of survival times** when truths are generated from a **Weibull model**: box plots of biases (upper panel) and binned RMSEs (lower panel) to compare the performance of ITE estimates under **balanced design**.

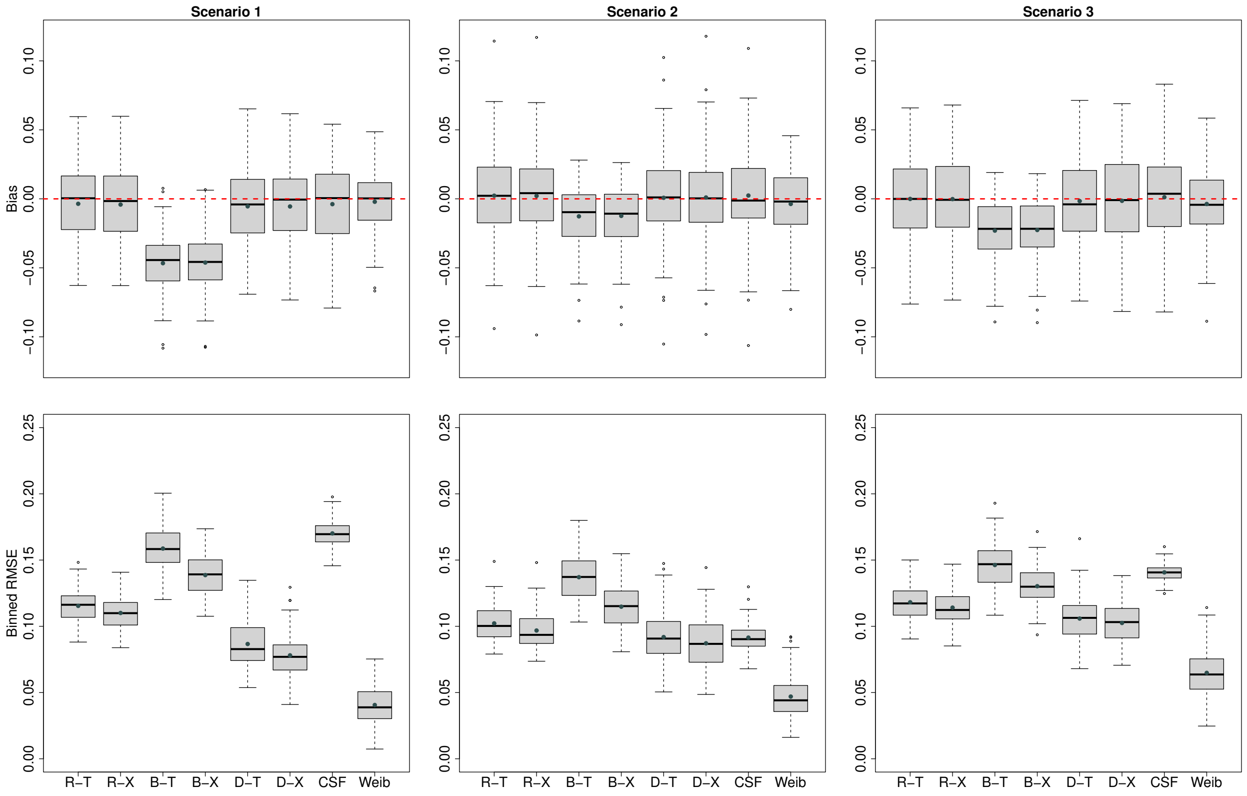

Web Figure 7: Simulation results at the **75% percentile of survival times** when truths are generated from a **Weibull model**: box plots of biases (upper panel) and binned RMSEs (lower panel) to compare the performance of ITE estimates under **dependent design**.

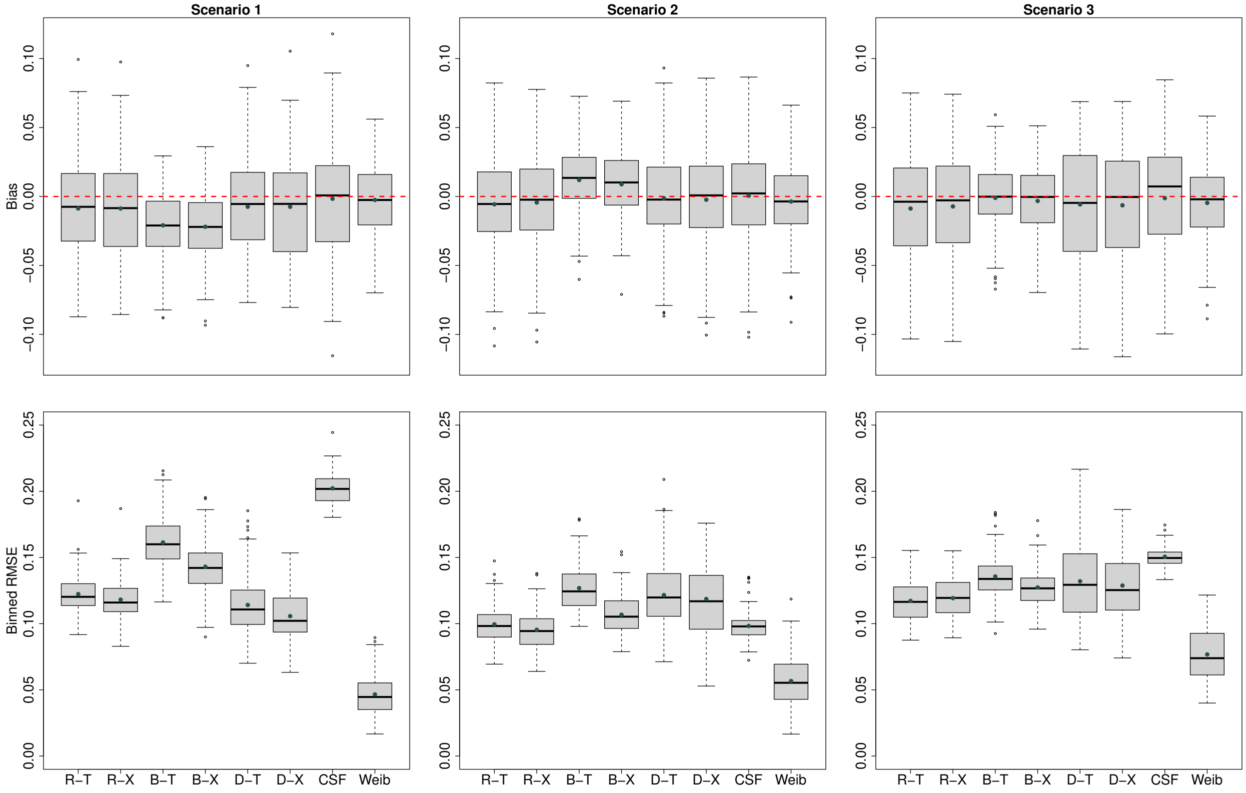

Web Figure 8: Simulation results at the **75% percentile of survival times** when truths are generated from a **Weibull model**: box plots of biases (upper panel) and binned RMSEs (lower panel) to compare the performance of ITE estimates under **unbalanced design** with sample size of 4000.

2 Appendix S2: Additional figures and tables for real data analysis

2.1 AREDS data analysis

Web Table 3 shows the summary of baseline characteristics of the AREDS data. Web Figure 9 plots the histograms of the estimated propensity scores for each treatment group in AREDS to check the unconfoundedness assumption.Web Figure 10 shows the mean treatment effect in RT and RC groups at year five on two other splits of data for each method. Web Figure 11 shows the mean treatment effect in RT and RC groups at year three for each method under three splits of data.

Web Table 3: Baseline characteristics of the AREDS data used in the analysis.

Number of subjects	All ($n = 806$)	Placebo ($n = 391$)	Antioxidants and Zinc ($n = 415$)	p-value*
Age				0.4905
Mean (SD)	68.77 (5.05)	68.90 (5.17)	68.66 (4.93)	
Median (Range)	68.60 (55.30-81.00)	68.50 (55.30-81.00)	68.70 (55.50-79.50)	
Sex (n, %)				0.8236
Female	466 (57.82)	224 (57.29)	242 (58.31)	
Male	340 (42.18)	167 (42.71)	173 (41.69)	
Smoking (n, %)				0.6877
Never Smoked	393 (48.76)	194 (49.62)	199 (47.95)	
Former/Current Smoker	413 (51.24)	197 (50.38)	216 (52.05)	
AREDS AMD categories (n, %)				0.5474
2	312 (38.71)	158 (40.41)	154 (37.11)	
3	457 (56.70)	214 (54.73)	243 (58.55)	
4	37 (4.59)	19 (4.86)	18 (4.34)	
Baseline AREDS AMD severity score				0.6303
Mean (SD)	4.09 (2.06)	4.13 (2.06)	4.06 (2.07)	
Median (Range)	4 (1-8)	4.00 (1-8)	4 (1-8)	

*p-value is based on two-sample t-test or Chi-square test for continuous or categorical variables.

2.2 AREDS2 validation data analysis

Web Figure 12 plots the Kaplan-Meier curves for the RT ("recommended for treatment") and RC ("recommended for control") cohorts in AREDS2 (where the treatment recommendation rule is based on the D-X result on AREDS data) when ITE is estimated at year five. It shows that the overall 5-year progression-free probability is significantly higher in the RT group than in the RC group in this AREDS2 dataset (log-rank test $p = 0.011$).

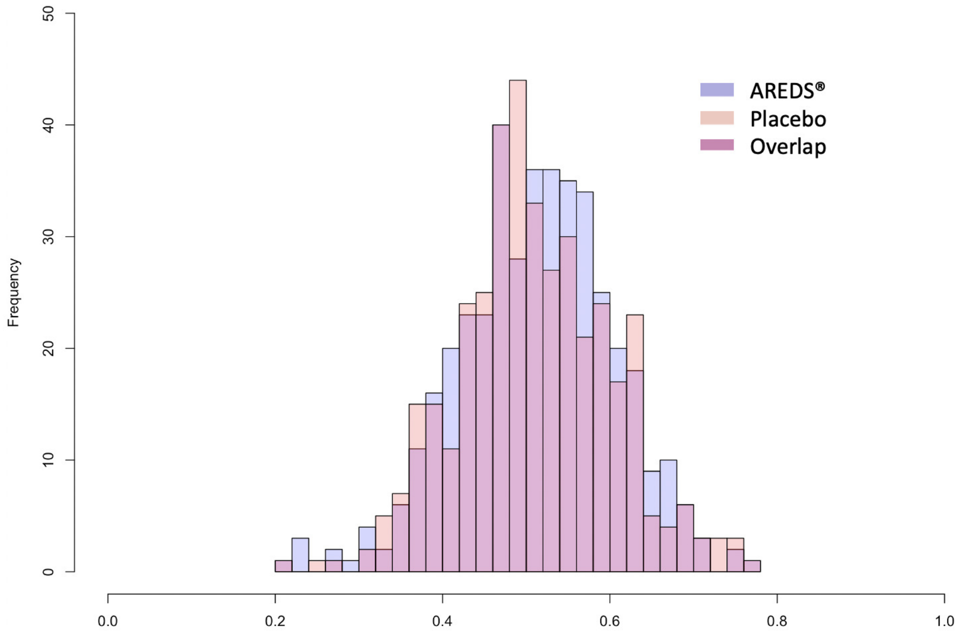

Web Figure 9: Histograms of the estimated propensity scores of getting AREDS formula in the AREDS dataset.

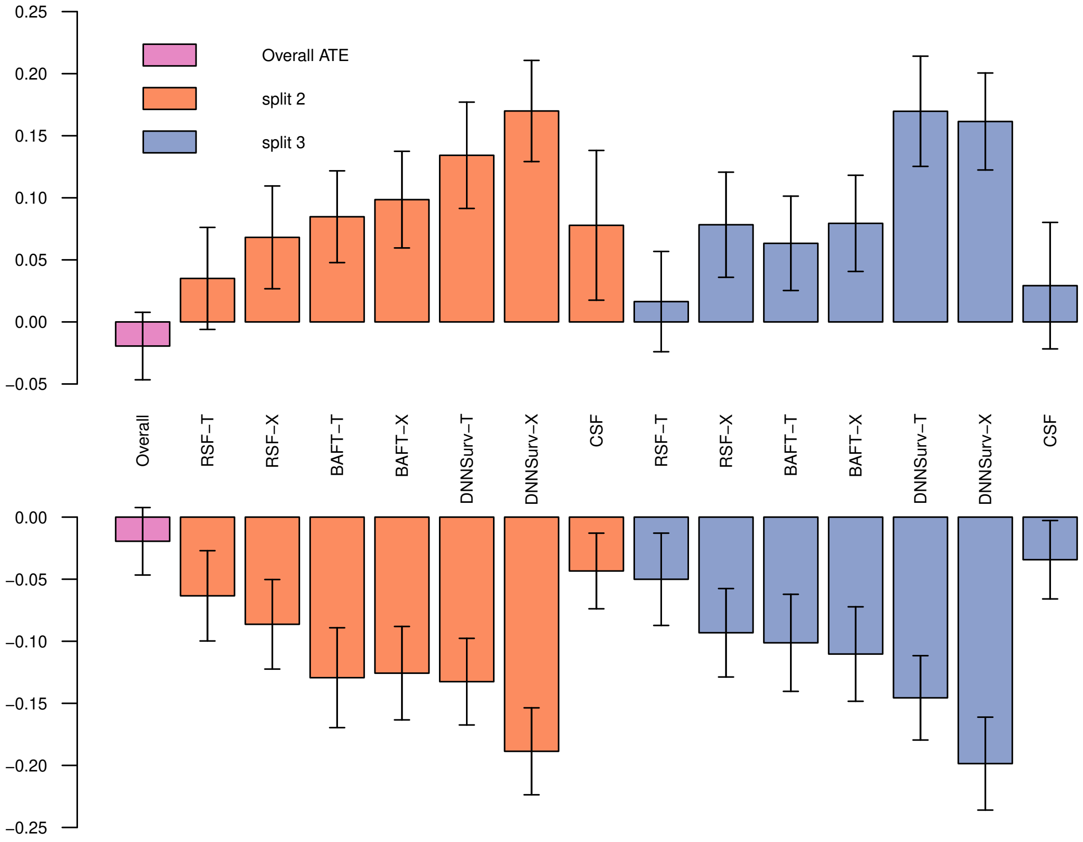

Web Figure 10: The mean treatment effect (KM estimates) at year five of participants in the RT cohort (recommended for taking the treatment) (upper panel) and in the RC cohort (recommended for taking the placebo) (lower panel) from each method in other two splits of data.

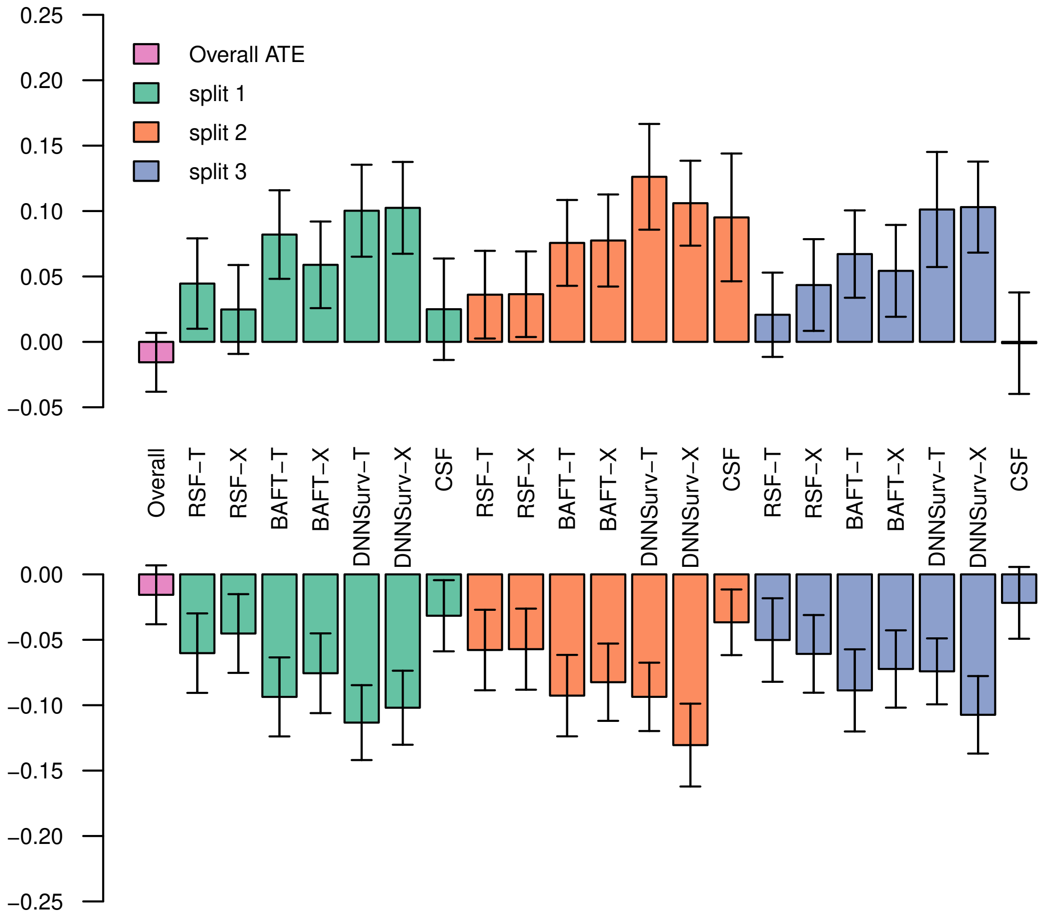

Web Figure 11: The mean treatment effect (KM estimates) at year three of participants in the RT cohort (recommended for taking the treatment) (upper panel) and in the RC cohort (recommended for taking the placebo) (lower panel) from each method in each split of data.

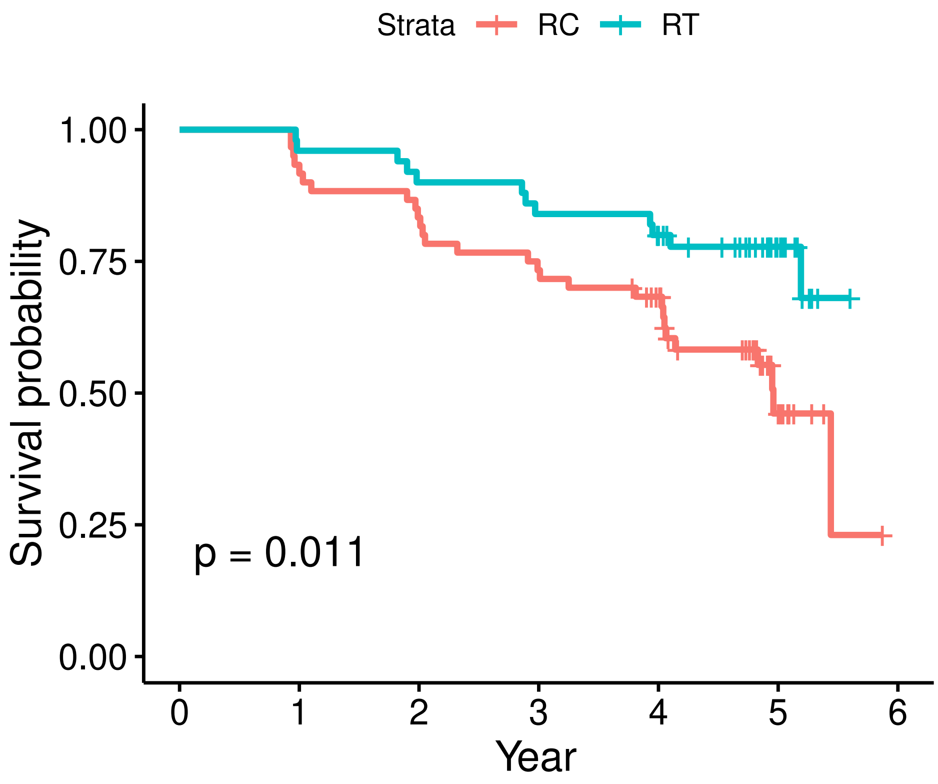

Web Figure 12: The Kaplan Meier curves of RT and RC cohorts in AREDS2 where the treatment recommendation rule is based on the D-X result on AREDS data at year five. Here ‘RT’ means ‘recommended for taking the AREDS formula’, and ‘RC’ means ‘not recommended for taking the AREDS formula’. The p-value is from the log-rank test.
